# Machine learning-based fetal health prediction and development of smart web application

**DOI:** 10.3389/frai.2026.1829833

**Published:** 2026-07-08

**Authors:** Chetan Puri, K. T. V. Reddy, Pradnyawant M. Gote

**Affiliations:** 1 Department of Computer Science and Engineering, Faculty of Engineering and Technology, Datta Meghe Institute of Higher Education and Research (DU), Wardha, Maharashtra, India; 2 Datta Meghe Institute of Higher Education and Research (DU), Wardha, Maharashtra, India; 3 Department of Computer Science and Design, Faculty of Engineering and Technology, Datta Meghe Institute of Higher Education and Research (DU), Wardha, Maharashtra, India

**Keywords:** deep learning, feature selection, fetal health, machine learning, web development

## Abstract

**Introduction:**

Fetal health monitoring is critical for early identification of pregnancy-related risks. Manual interpretation of cardiotocography (CTG) signals is subjective and variable among healthcare professionals.

**Methods:**

A machine learning-based framework was developed to classify fetal health into Normal, Suspect, and Pathological categories using CTG-derived clinical features. The dataset was preprocessed through duplicate removal, normalization, class balancing using SMOTEENN, multicollinearity analysis via VIF, and Kruskal–Wallis statistical feature selection. Eleven machine learning and neural network models were trained and compared, including Logistic Regression, K-Nearest Neighbors, SVM, Decision Tree, Random Forest, Gradient Boosting, AdaBoost, XGBoost, LightGBM, Multi-Layer Perceptron, and Deep Neural Network.

**Results:**

LightGBM achieved the best overall performance with 96.03% accuracy, 91.99% balanced accuracy, 93.05% macro F1-score, 99.02% ROC-AUC, 88.91% Cohen's Kappa, and 89.01% MCC. SHAP-based explainability identified abnormal short-term variability and fetal heart rate accelerations as the most important features.

**Discussion:**

The best-performing LightGBM model was integrated into a Streamlit-based web application for real-time fetal health prediction, demonstrating its potential as a clinical decision-support tool.

## 1 Introduction

Maternal and fetal health monitoring is the backbone of all practices when it comes to pregnancy and childbirth, which includes fetal monitoring and prenatal diagnostics. These diagnostic techniques help in identifying intrinsic problems and also prevent stillbirths, ensuring safe delivery. Over the last years, more and more with medical technologies have been focused on prevention rather than purely reactive treatments, most notably in the field of fetal health, since early detection can substantially increase pregnancy results (Liston et al., 2007). Cardiotocography (CTG), ultrasound, and biochemical screening are the most common conventional monitoring methods used for fetal assessments. Despite relying heavily on healthcare experts, their readings and understandings can be dependent and variable. This can lead to different outcomes due to subjective perspectives (Ponsiglione et al., 2021; Nazli et al., 2025).

Fetal monitoring is well-recognized as a significant aid in the diagnosis and prevention of complications such as intrauterine growth restriction (IUGR), fetal distress, and labor-related suffering, which are among the leading causes of neonatal morbidity and mortality in many countries (Lawn et al., 2011). Approximately 2.4 million newborns died within the first month of life in the year 2019, as per the World Health Organization (WHO). Many of these deaths could potentially be prevented if proper diagnoses were made during pregnancy itself (Saha, 2023). Even though many state-of-the-art technologies present in urban hospitals, rural and low-resource settings still face problems due to a dearth of qualified professionals, deficient infrastructure, and limited availability of good parental care. A landmark 2025 scoping review of 39 systematic and narrative reviews, spanning reproductive, prenatal, postpartum, and neonatal AI applications, concluded that home-monitoring platforms for pregnant women are associated with 7–11% reductions in maternal mortality and preeclampsia incidence, yet underscored that most evidence still derives from retrospective, single-center studies with limited external validation (Somerville and Abuadas, 2025).

**Figure 1:**
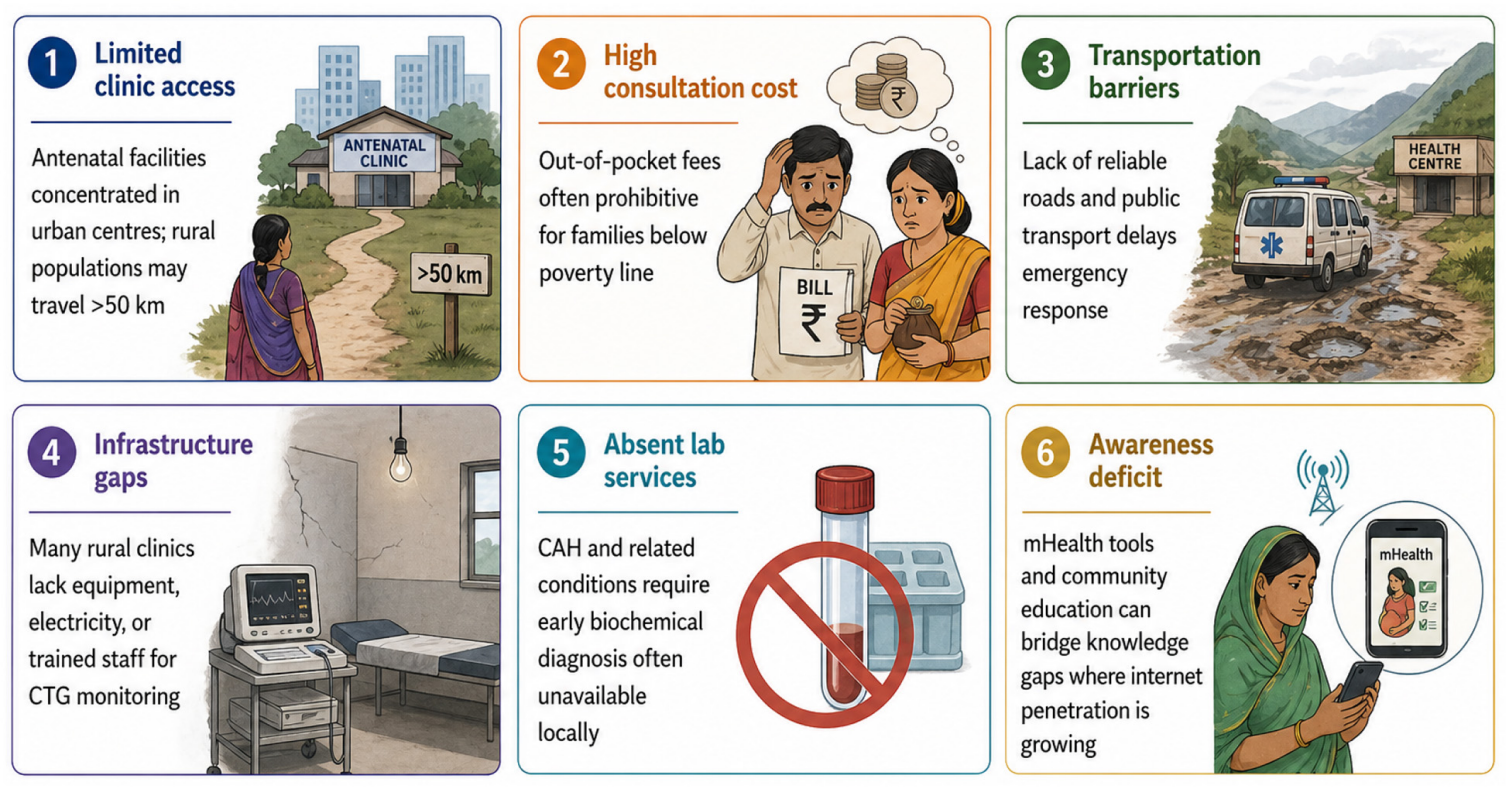
Barriers to prenatal care in low-resource settings identified from Heaman et al. (2015), Gangolli et al. (2005), and UNICEF Indonesia (2017).

These gaps in healthcare delivery are pronounced in underserved areas, wherein expectant mothers often encounter obstacles such as limited access to antenatal clinics, transportation barriers, high consultation fees and a general lack of awareness (Heaman et al., 2015). Most maternal and neonatal deaths happen in low-resource settings where laboratory services are lower or absent, presenting with CAH as one of the most common abnormalities requiring early diagnosis to avoid prenatal death (UNICEF Indonesia, 2017). The density of healthcare providers is worryingly low in many low-income countries, leading to overworked clinics and poor fetal evaluations. Additionally, where services are available, traditional diagnostic tools may not be used appropriately because of the lack of trained personnel or lengthy decision-making pathways (Gangolli et al., 2005). These major barriers to prenatal care in low-resource settings are illustrated in Figure 1.

Machine learning (ML) applications in peripartum care have expanded considerably. Steinberg et al. (2025) conducted a comprehensive scoping review of ML in peripartum settings, drawing on MEDLINE, Cochrane, EMBASE, and confirmed that ML tools demonstrate strong predictive potential for early intervention and clinical decision support, while also cautioning that solutions must be transparent, robust, and adaptable to diverse healthcare systems to achieve equitable global impact. These observations support the reasoning behind creating the light and web-accessible model in the present study. Machine learning algorithms for early diagnosis and clinical detection of pre-eclampsia were also developed by Jain et al. (2025), and support the expanding utility of machine learning for predictive risk and support in maternal-fetal health.

One of the main structural problems within CTG data is class imbalance, where pathological records represent less than 10% of total records. In February 2026 Hawrami, Cengiz, and Dnder (Hawrami et al., 2026) conducted a first true systematic comparison on five resampling methods (including SMOTE, ADASYN, SMOTEENN, etc.) across seven classifier families with imbalance-sensitive measures. They found that a hybrid SMOTEENN method, such as is used in this work, is beneficial, and standard accuracy measures do not work for classifying medical CTG, and that the correct choice of resampling method can significantly affect classifier validity for the minority (pathological) classes.

**Figure 2:**
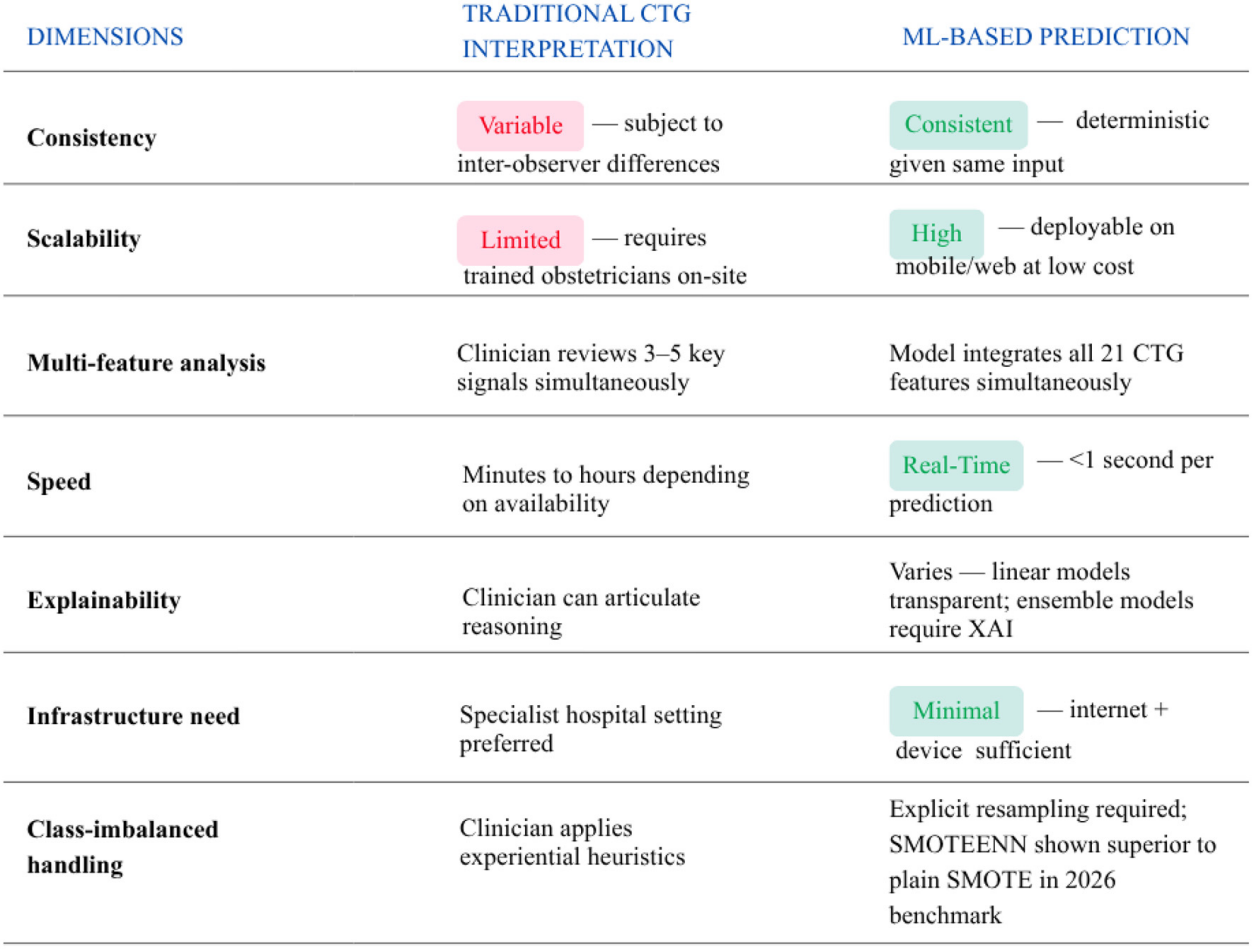
Traditional vs. ML-based fetal health assessment: Comparison synthesized from Bertini et al. (2022), Ponsiglione et al. (2021), Mechael (2009), and Hawrami et al. (2026). ML advantages are most pronounced in resource-limited settings.

In the future, artificial intelligence (AI) and machine learning (ML) could play significant roles in how we care for pregnant women. These technologies may bring about a revolution in prenatal care. Here, several tasks focus on employing machine learning (ML) to predict and organize data, as well for pattern recognition, which in turn allow machines to learn from historical data and assist health professionals in making decisions (Bertini et al., 2022). Autonomous fetal health prediction through ML can curb human errors, make the diagnosis more accurate, and consequently assist in raising alarms for risky pregnancies even earlier. These types of systems can compare the rhythm of a baby's heartbeat, contractions of the uterus, a mother's vital signs, and other physical signs that might not be noticed by human observers (Malviya and Goyal, 2023). A comparative overview of traditional and ML-based fetal health assessment methods is presented in Figure 2.

One significant benefit of these data-driven systems is that they are amenable to being applied in various clinical settings, from rural to urban clinics or from hospitals to a mobile environment without the need of any significant infrastructure. Once these algorithms are developed, they can operate in low cost mobile devices and used to detect risk and monitor patients in low-resource settings (Mechael, 2009). Importantly, ML algorithms can be integrated into mobile or web-based healthcare support platforms, providing remote guidance to healthcare workers, community health volunteers, or expectant mothers themselves. This is especially relevant in countries like India, where mobile health (mHealth) applications are already playing a role in expanding access to healthcare information (Islam et al., 2022).

Numerous investigations have shown that machine learning (ML) could be beneficial in predicting a fetus's health. For example, applying techniques such as Support Vector Machines (SVM), Random Forests (RF), K-Nearest Neighbors (KNN), and neural networks to CTG data has produced positive results in categorizing fetal health conditions as normal, suspect, or pathological (Mennickent et al., 2023). These machine systems usually outperform typical statistical techniques in terms of precision, and they can get even better as they are given more information to work with throughout their operation. Additionally, they can also be created to consider multiple risk factors at once, like a woman's age, blood pressure, and blood sugar levels, past pregnancy complications, and environmental influences allowing for a more comprehensive assessment of fetal and maternal health (Yang, 2022).

But before that vision can be realized, there are several challenges associated with the implementation of ML in prenatal diagnostic tools. One of the major challenges is model interpretability. To build the credibility of predictions produced by these systems, healthcare providers must trust neural network algorithms, and they require an architectures capable of explaining model decisions, i.e., Explainable Artificial Intelligence (XAI) (Liu and Panagiotakos, 2022). The quality of input data is equally important. Many algorithms are trained on clean and well-standardized datasets, which may be differ significantly from the noisy and incomplete data commonly found in clinical environments. Therefore, algorithms should be robust, capable of generalizing effectively, and able to handle uncertain or missing data inputs (Benfradj et al., 2024).

Cost-effectiveness is yet another important factor to consider when selecting the proposed solutions. Artificial intelligence (AI) and machine learning (ML) provide the highest accuracy and efficiency, but they demand powerful computational resources along with domain expertise in the development, training, and maintenance of these systems. Therefore, to ensure large-scale adoption in low-resource settings, there is a need for design lightweight, efficient and interpretable algorithms (Putra et al., 2024). This can further help in reduce the costs and lessen dependency on centralized healthcare facilities by taking advantage of cloud-based services or on-device processing for real-time prediction (Dlugatch et al., 2023).

Besides this, it is important to consider ethical and legal matters when using AI in vital sectors like maternal and newborn care. This includes factors such as patient privacy (Topol, 2019), informed consent (Morley et al., 2020), algorithmic bias (Obermeyer et al., 2019) and accountability (Floridi et al., 2018). For instance, an inaccurate prediction of fetal distress could be critically detrimental to patient management and health outcomes (Challen et al., 2019), hence requiring these systems to be developed on the premises of fairness, robustness and integrity (Jobin et al., 2019).

The aim of this study is to develop and evaluate machine learning (ML) and neural network based algorithms for accurate fetal health prediction from CTG data. Specifically, this study evaluates the performance of 11 algorithms: Random Forest, SVM, Decision Tree, Logistic Regression, LightGBM, Extreme Gradient Boosting (XGBoost), AdaBoost, Gradient Boosting, K-Nearest Neighbors, Multi Layer Perceptron, and Deep Neural Network (DNN) on a publicly available CTG dataset. Performance is assessed based on accuracy, balanced accuracy, interpretability, and scalability. Additionally, we demonstrate the practical deployment of the best performing model through a user friendly web application to assist healthcare providers and expectant mothers in real time fetal health monitoring.

## 2 Related work

**Figure 3:**
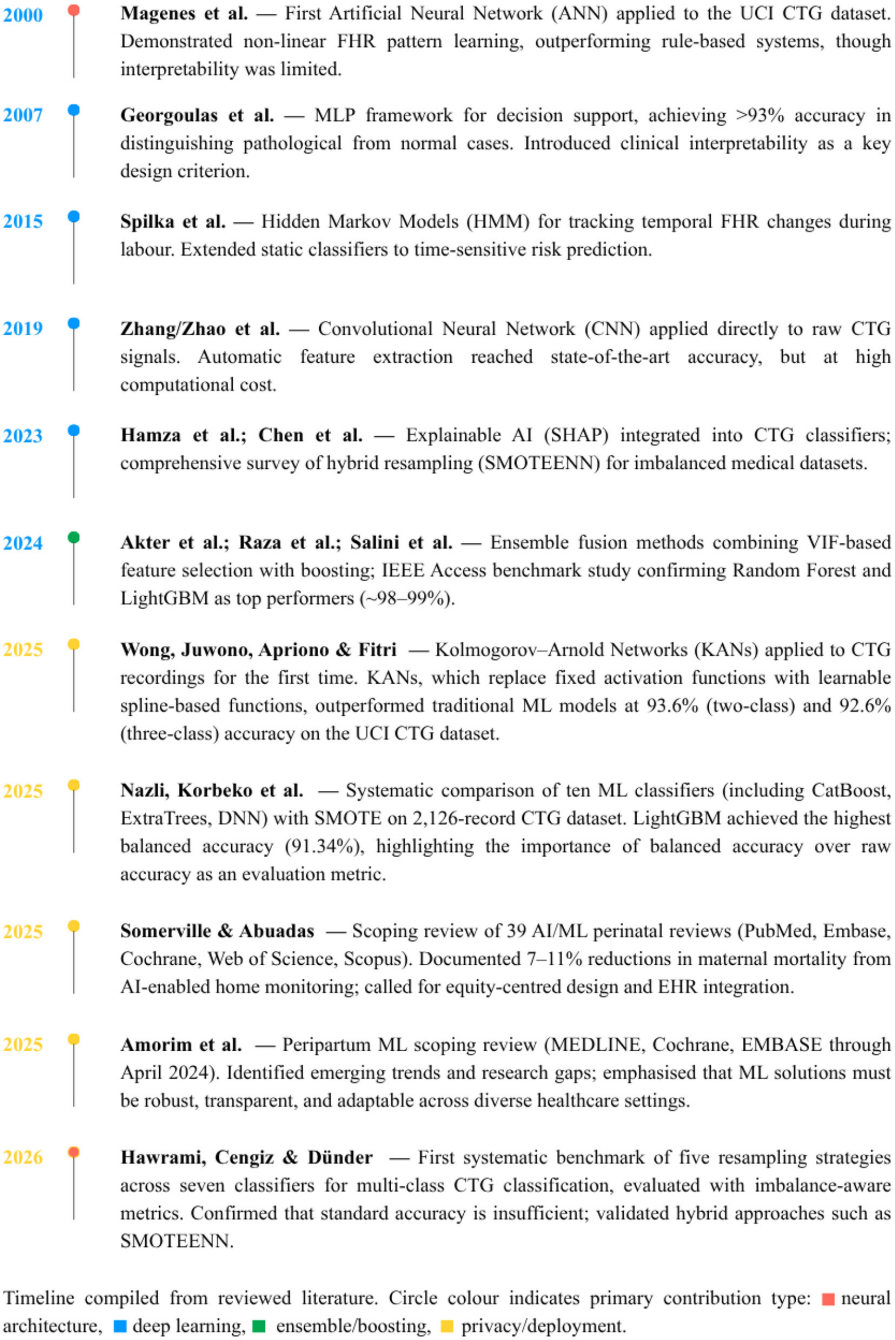
Key milestones in ML-based fetal health research.

Machine learning has been the focus for the classification of CTG-based fetal health for more than 20 years. The Cardiotocography (CTG) dataset, acquired from the UCI Machine Learning Repository (Dua and Graff, 2019), have been extensively used as a benchmark, which consisted of physiological characteristics of FHR and UC signals, classified as normal, suspect, and pathological. Figure 3 provides a summary of major achievements.

Magenes et al. (2000) were among the pioneers in using Artificial Neural Networks for CTG classification. They demonstrated that neural networks could model non-linear associations within FHR and UC signals. These first algorithms were significant but were not highly accurate nor were they clinicaly interpretable. Further works were developed on this, such as Georgoulas et al. (2007) whose approach based on a grammatical evolution technique obtained 93% accuracy in distinguishing between a normal and a pathological cases.

Support Vector Machines were also considered in the context of a high dimensional feature space. Spilka et al. (2015) used sparse feature space trajectory analysis in conjunction with an SVM classifier for classification of intrapartum FHR signals. This was a progression away from static classification toward a more dynamic, temporal approach. Random Forest classifiers were discussed by Salini et al. (2024) who were able to obtain a classification accuracy on the CTG dataset of nearly 98.45%; Random Forests additionally provide feature ranking, which improves their transparency.

Zhao et al. (2019) presented an approach to analyze raw CTG signal directly, called DeepFHR, a CNN based system that performed automatic feature extraction and acquired a high level of accuracy; however, this comes at the price of a very high computational cost and is thus poorly suited for low computational devices commonly encountered in the clinical settings. Biswas et al. (2025) proposed hybrid machine learning algorithms for ECG-based arrhythmia detection using autoencoder and convolution features. This is in agreement with our research thatdeep feature extraction and machine learning combined can enhance biomedical signal classification.

On the ensemble methods side, Al Duhayyim et al. (2023) designed an ensemble that combine Decision Tree, Logistic Regression, and Gradient Boosting through voting. They reported that F1 performance and tolerance to imbalanced data were higher with ensemble method. More recently, Akmal et al. (2023) and Kapila and Saleti (2023) found that careful data preprocessing and ensemble approach can generally achieve good performance for classification on the CTG dataset. The paper by Chhabra et al. (2026) used EEG for emotion detection, which combined the chaotic local binary pattern (CLBP) features with ensemble method. Although their data were not CTG signals, the combination of advanced feature extraction methods with ensemble algorithms showed its effectiveness for classification tasks in biomedical signals.

Dealing with the natural class imbalance of normal vs. pathological CTG records remains one of the most outstanding problems of the domain. Nazli et al. (2025) compares 10 classifiers (including CatBoost, ExtraTrees, ANN, and DNN) with SMOTE oversampling using the standard 2,126 record CTG dataset and obtained LightGBM with the best balanced accuracy (91.34%) which, as suggested, is the clinically relevant measure over raw accuracy with imbalanced data. In addition, Hawrami et al. (2026) reported the first direct comparison of the five resampling methods on seven classifier families where combined (hybrid) resampling methods like SMOTEENN generally yielded the best results compared to single resampling methods with imbalance aware measures. It has also been highlighted in Altalhan et al. (2025) by over 68 publications and several survey papers reviewing various types of oversampling, undersampling and combined methods on medical classification problems and suggests combined methods when a severe class imbalance occurs.

In recent years feature selection has also begun to draw more interest. The study proposed by Akmal et al. (2023) combined the Recursive Feature Elimination (RFE) approach with Lasso regression to find the minimum set of input features while retaining its accuracy. This makes the final algorithms ideal to be implemented on a mobile device or any resource-limited device.

Another key architectural change came from Wong et al. (2025) who applied Kolgorov-Arnold networks (KANs) to the CTG classification problem, as shown in Figure 3. Unlike standard networks which use fixed activation functions, KANs use learned splines for activations along the network edges and achieve 93.6% accuracy on binary classification, and 92.6% on three-class classification. In comparison to standard deep networks KANs do offer more interpretability, however accuracy for three-class classification has plenty of room to improve.

The demand for clinical explainability has led to an alternative but equally significant area of research. Holzinger et al. (2017) highlighted the importance of making black-box algorithms provide explainable models even if there is high accuracy as clinicians have to trust these models. The applicability of SHAP-based explainable AI was demonstrated on CTG data for fetal health classification, where the paper clearly showed that predictable output of models helps clinicians and a prediction that is understandable has a clinical application. Even further, Innab et al. (2024) incorporated SHAP explanation into a LightGBM pipeline for the management of fetal and maternal health and presented that high accuracy and clinical explainability are not necessarily contradictory.

With respect to the data quality and patient privacy, Mondal et al. (2025) designed reliable preprocessing algorithms to clear the artifact and fill in the missing values from the CTG signals, which is necessary in the countryside because of the possible instability of the equipment used to record the CTG signals. For the purposes of data privacy, Nguyen et al. (2022) adopted a federated learning approach that enables algorithms to train themselves across multiple institutes without exchanging patient data to any central server.

On the deployment side, Mapari et al. (2024) established proof-of-concept for the use of AI tools for remote maternal care in rural areas. A 2025 scoping review by Somerville and Abuadas (2025) that synthesized 39 review-level studies found that AI-enhanced remote monitoring systems can provide significant improvements in maternal and fetal mortality in certain contexts, and recommended the need for prospective multi-center validation, auditable fairness analyses, and integration with electronic health records (EHR) prior to general clinical deployment. These latest research results indicate that the ML-based fetal monitoring solutions are developing mature and continue effort is needed for those challenge areas, such as class imbalance, feature selection, model explainability.

## 3 Methodology

**Figure 4:**
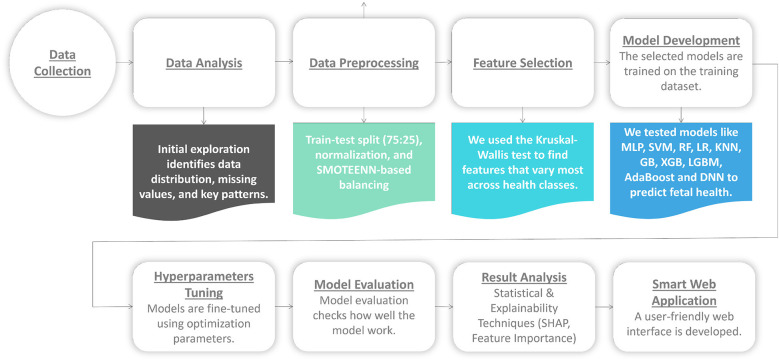
Workflow of the proposed methodology process.

In this study, our work focuses on the application of machine learning (ML) and neural network-based methods to predict fetal health status as shown in Figure 4. The overall objective is to develop an intelligent, data-driven decision-support system capable of automatically classifying fetal health status into Normal, Suspect, or Pathological categories based on features extracted from CTG signals. For this purpose we use well-established supervised learning algorithms and sophisticated neural networks architecture which are well- recognized for their pattern recognition performance in biomedical context.

### 3.1 Data collection

For this study, the dataset from the UCI Machine Learning Repository was used (Dua and Graff, 2019). The UCI Machine Learning Repository is a popular data sharing space, with a variety of datasets available for data mining and machine learning purposes. The dataset chosen for this project was the fetal health classification dataset, which comprised clinically approved and pre-processed features that have been collected using cardiotocography (CTG) recordings. The dataset comprises 2,126 records, each containing 21 numeric input features and one categorical target variable (fetal_health), resulting in 22 attributes per record. The input features include fetal baseline heart rate, accelerations, fetal movements, uterine contractions, and variability metrics (both short-term and long-term). Each record is labeled with one of three fetal health classes: Normal, Suspect, or Pathological, as shown in Table 1.

**Table 1:** Characteristics of fetal health data set including typical value ranges.

No.	Feature	Description	Range
01	Baseline value	Mean FHR during stable periods (bpm)	80–180 bpm
02	Accelerations	Temporary increases in FHR above baseline	0.0–1.0
03	Fetal movement	Number of detected fetal movements	0.0–1.0
04	Uterine contractions	Number of uterine contractions	0.0–1.0
05	Light decelerations	Minor decreases in FHR from baseline	0.0–1.0
06	Severe decelerations	Significant drops in FHR	0.0–1.0
07	Prolonged decelerations	Extended periods of low FHR	0.0–1.0
08	Abnormal short-term Variability	Percentage of Abnormal FHR Variability	0–100%
09	Mean value of short-term variability	Average of short-term FHR changes	0–7 ms
10	Percentage of time with abnormal long-term variability	Proportion of abnormal long-term FHR (%)	0–100%
11	Mean value of long-term variability	Average of long-term FHR variations	0–50 ms
12	Histogram width	Range of FHR values observed	0–200 bpm
13	Histogram min	Lowest FHR value recorded	50–150 bpm
14	Histogram max	Highest FHR value recorded	100–200 bpm
15	Histogram number of Peaks	Count of peaks in the FHR distribution	0–20
16	Histogram number of Zeroes	Times when no FHR was detected	0–20
17	Histogram mode	Most frequent FHR value	60–180 bpm
18	Histogram mean	Average of all FHR values	60–180 bpm
19	Histogram median	Median FHR value	60–180 bpm
20	Histogram variance	Spread/variability of FHR values	0–3000
21	Histogram tendency	Direction of FHR trend (increasing or decreasing)	−1, 0, 1
22	Fetal health	Health status: normal, suspect, pathological	1–3 (categorical)

### 3.2 Data analysis

In this phase, we conducted a comprehensive exploratory data analysis of 2,126 cardiotocographic (CTG) records labeled into three classes: Normal, Suspect, and Pathological.

**Figure 5:**
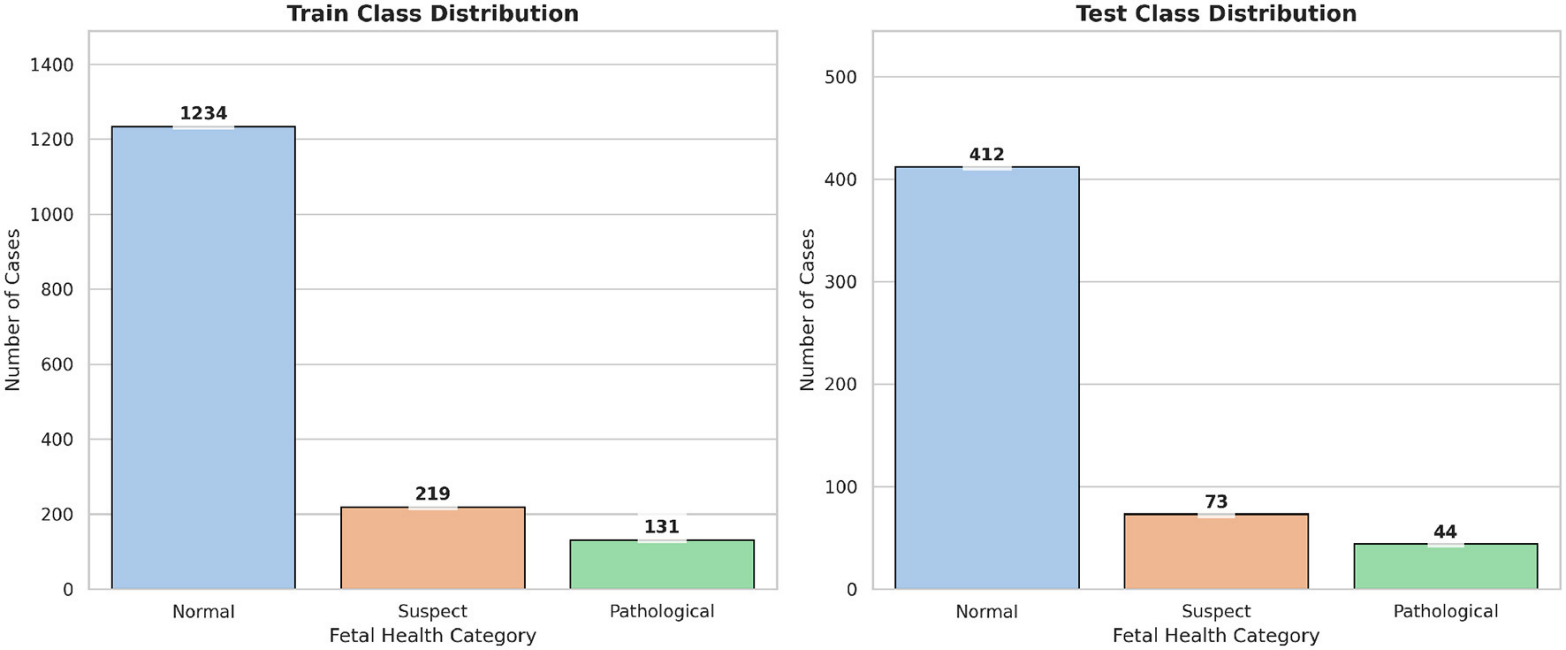
Training and testing data set distributions for machine learning and neural networks based algorithms.

As shown in Figure 5, the class distribution analysis revealed a significant imbalance, with the majority of samples belonging to the Normal class, followed by the Suspect and Pathological classes. This skewed distribution increases the risk of classification bias, particularly affecting the performance on minority classes.

**Figure 6:**
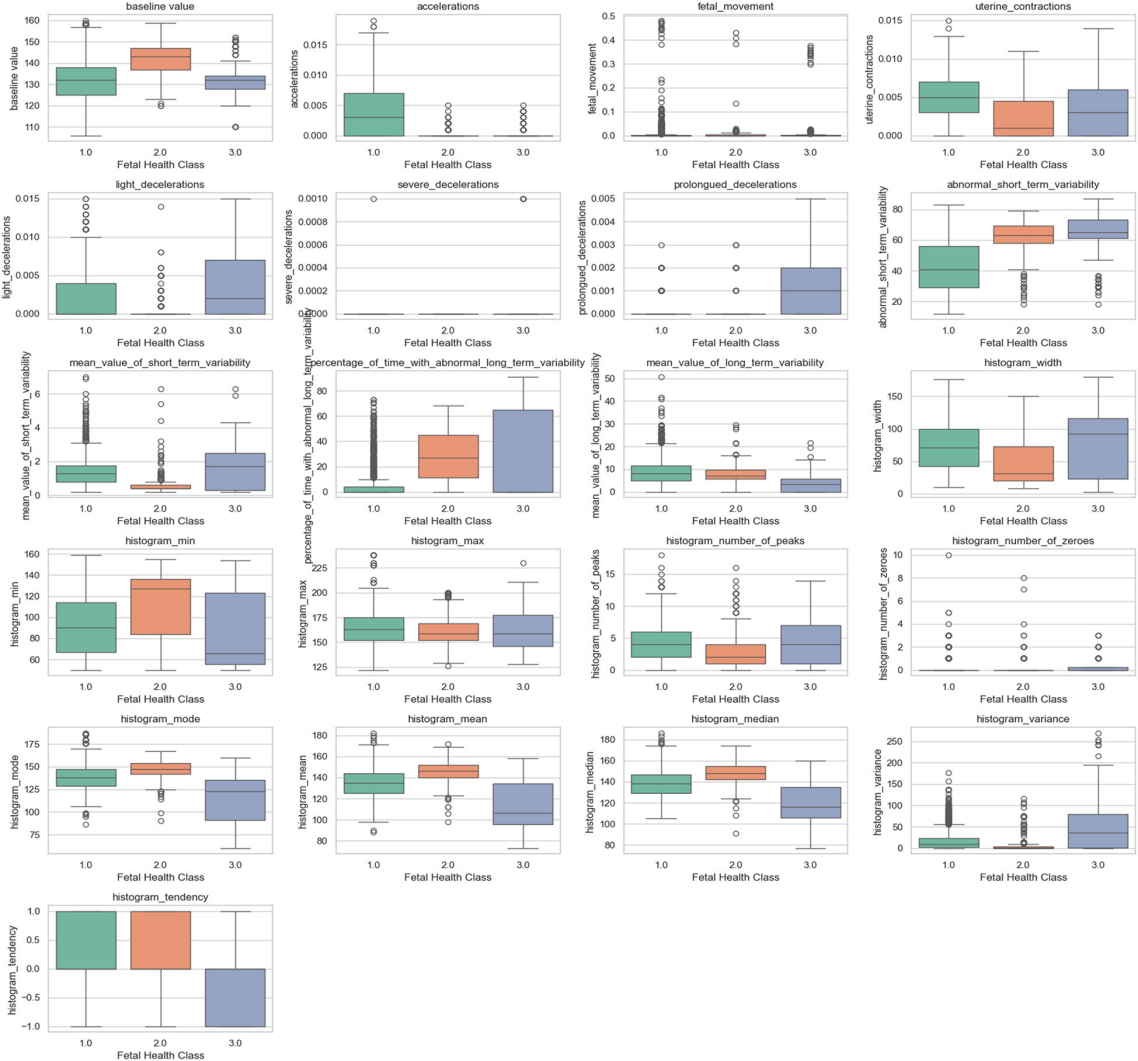
Distribution of each features and outliers for three fetal classes (Class 1: Normal, Class 2: Suspect, Class 3: Pathological) are visualized by Boxplots.

**Figure 7:**
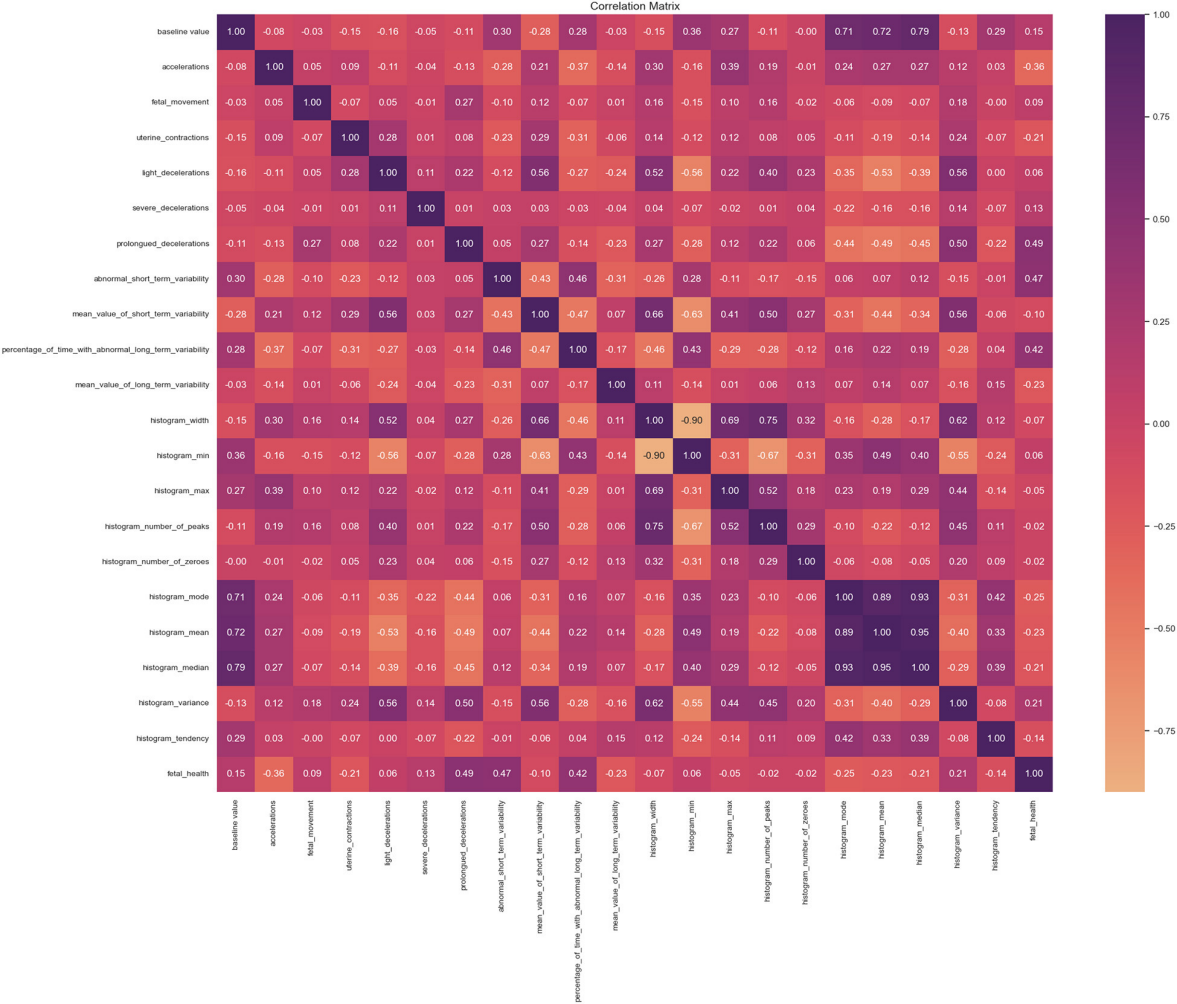
Correlation heatmap illustrating pairwise relationships among all 22 CTG attributes.

To investigate feature relationships, further exploratory data analysis (EDA) was conducted using box plots, as shown in Figure 6, and a correlation heatmap, as shown in Figure 7. The correlation matrix revealed that several features exhibited moderate to high correlations. For instance, abnormal short-term variability (ASTV) showed a strong correlation with mean short-term variability (MSTV). These findings guided the feature selection and dimensionality reduction strategies.

### 3.3 Data pre-processing

The first step of the methodology is data preprocessing and exploratory data analysis (EDA). A publicly available CTG dataset consisting of 22 attributes related to fetal heart rate and uterine contraction signals, along with fetal health class labels, was used, as discussed in Section 3.1. Data preprocessing included missing data imputation, data normalization, encoding of class attributes, and visualization of attribute distributions. Correlation analysis and feature selection were then performed to reduce dimensionality and retain the most informative attributes.

#### 3.3.1 Handling of missing and duplicated values

During the step of preprocessing we found out 13 duplicates within the dataset. Duplicates within the dataset cause a bias and leads to overestimation of model performance, while decreasing the generalization ability. Consequently, the found duplicates were removed to ensure that training instances are distinct and statistical relevance of the dataset would be maintained.

#### 3.3.2 Removal of less informative features

As part of the data preprocessing pipeline, the correlation matrix was analyzed to identify and remove highly correlated or redundant features, thereby reducing multicollinearity and improving model generalization.

Based on the correlation analysis shown in Figure 7, five features were identified for removal: (1) Fetal Movement, (2) Severe Decelerations, and (3) Histogram Number of Zeroes showed very weak correlation with the target variable (fetal health outcome); (4) Histogram Median and (5) Histogram Mode exhibited strong correlation with other histogram-based features, particularly Histogram Mean, indicating redundancy. Removing these features reduced the feature space from 21 to 16 input variables while retaining variables with stronger predictive relevance and minimizing feature overlap.

#### 3.3.3 Feature normalization

**Figure 8:**
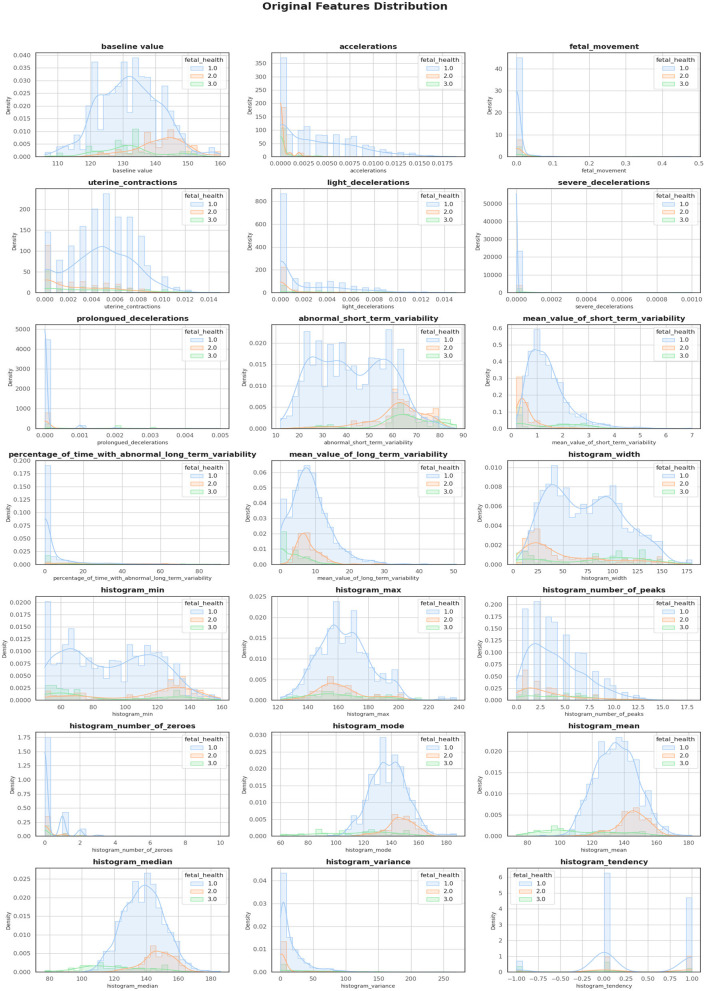
Original distributions of all features across the whole dataset without any resampling and normalization.

**Figure 9:**
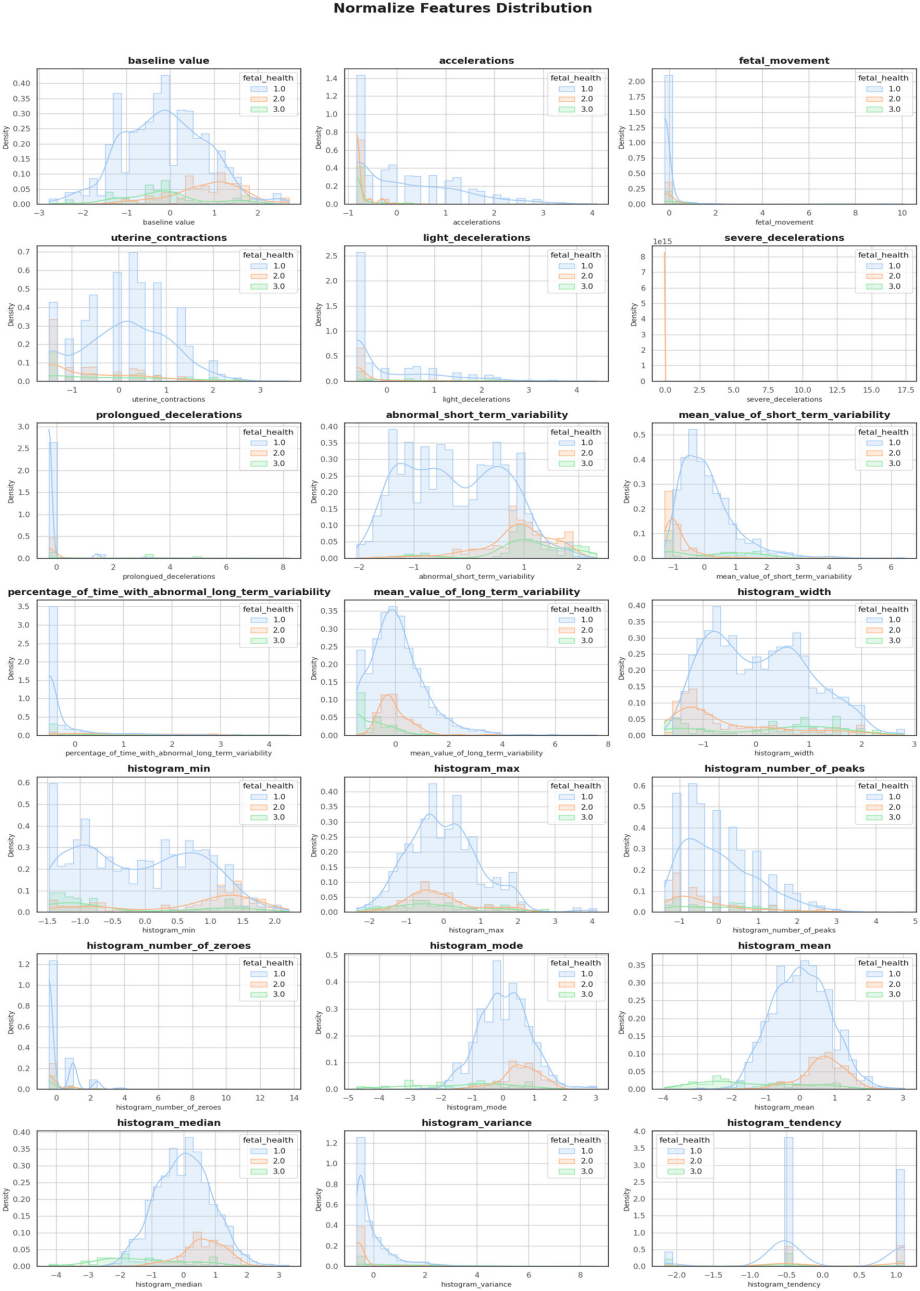
Normalized distribution of all features after applying StandardScaler feature scaling technique.

Feature scaling was performed to ensure that all input variables were represented on a comparable scale, preventing any feature from disproportionately influencing the learning process. We applied StandardScaler, which standardizes each feature by subtracting its mean and dividing by its standard deviation, as illustrated in Figures 8, 9. This preprocessing step is particularly important for scale-sensitive algorithms such as SVM, KNN, Multi-Layer Perceptron (MLP), and DNN, where differences in feature magnitude can bias distance calculations, gradient updates, and overall model performance.

### 3.4 Class imbalance handling

Class imbalance was one of the key issues observed in the fetal health dataset. The majority of records were assigned to the Normal class (Class 1), while the Suspect (Class 2) and Pathological (Class 3) categories contained far fewer samples. If this imbalance is not handled properly, the model may learn the majority-class pattern more strongly and produce high overall accuracy while performing poorly on the clinically important minority classes.

Figure 9 presents the class distribution before applying any balancing method. To avoid data leakage, the dataset was first split into training and testing sets. In the training set, the Normal class contained 1,234 samples, whereas the Suspect and Pathological classes contained 219 and 131 samples, respectively. The same imbalance was also present in the test set, with 412 Normal, 73 Suspect, and 44 Pathological cases. Therefore, class balancing was applied only to the training set, while the original test distribution was preserved for unbiased evaluation.

#### 3.4.1 SMOTEENN

A key challenge in fetal health prediction is class imbalance, where most samples belong to the Normal class, while comparatively fewer instances are labeled as Suspect or Pathological. This under-representation of clinically critical minority classes can bias learning algorithms toward the majority class, leading to reduced precision, recall, and overall classification performance for minority fetal health conditions. To address this issue, we incorporate SMOTEENN (Synthetic Minority Oversampling Technique combined with Edited Nearest Neighbors), a hybrid resampling method that first oversamples minority class instances using SMOTE and then applies the Edited Nearest Neighbors (ENN) cleaning algorithm to remove noisy or misclassified samples near the decision boundary.

**Figure 10:**
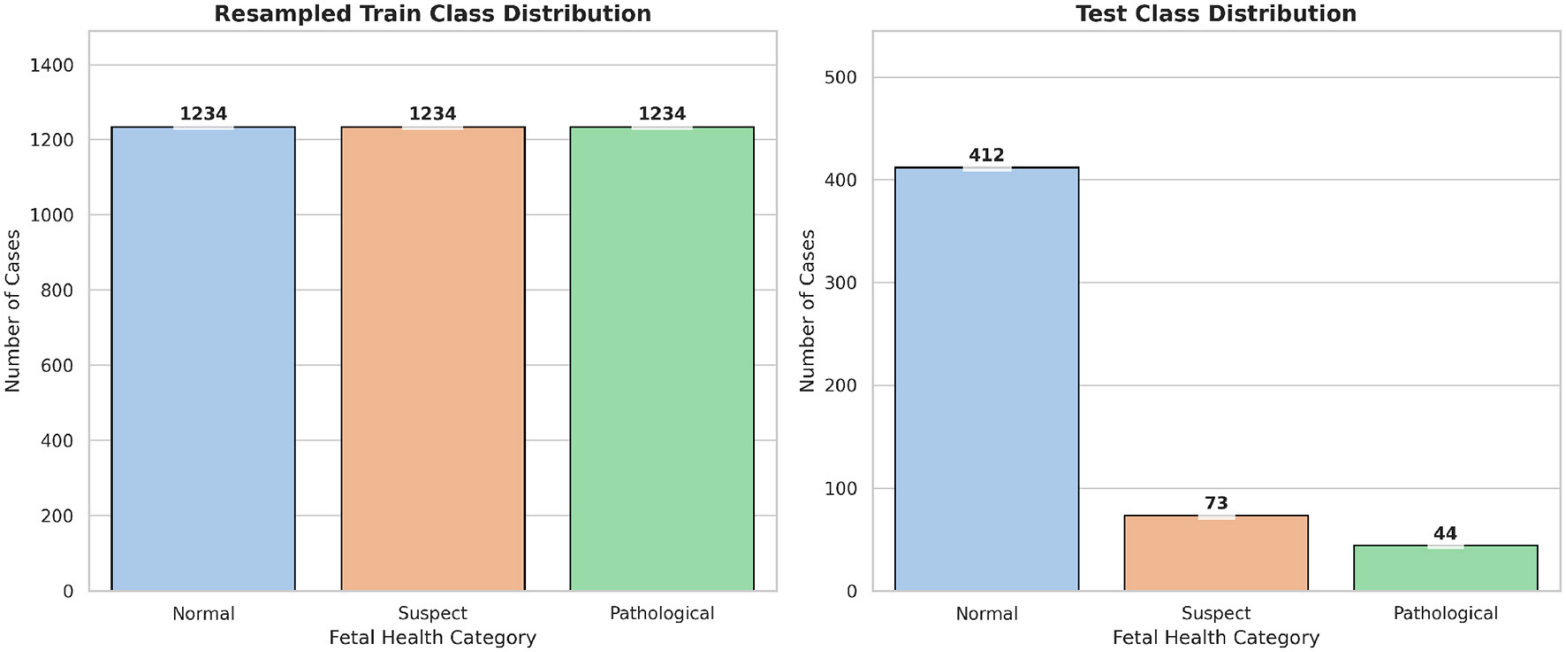
Training and testing dataset distributions after applying SMOTEENN.

Using this approach, SMOTEENN improves the models ability to learn patterns from the minority class, leading to improved predictive performance and more equitable outcomes in machine learning and neural network tasks. Subsequently, synthetic data was produced using the SMOTEENN method on the train dataset due to class inequality, and the equalized distribution was shown in Figure 10.

Table 2 summarizes the class distribution before and after applying SMOTEENN, and Figures 9, 10 visualize this transformation. Initially, the training set contained 1,234 Normal samples, 219 Suspect samples, and 131 Pathological samples, totaling 1,584 instances. This corresponds to 77.9% Normal, 13.8% Suspect, and 8.3% Pathological samples, indicating a clear class imbalance in the training data. After applying SMOTEENN exclusively to the training set, the resampled dataset contained 1,234 samples per class, yielding 1,234 Normal, 1,234 Suspect, and 1,234 Pathological samples. SMOTE generates synthetic samples for the under-represented classes, namely Suspect and Pathological, while ENN removes overlapping, noisy, or borderline samples to improve class separation. Since the balancing technique was applied only to the training set, the test set distribution remained unchanged to ensure an unbiased final evaluation. The resulting resampled training set contained 3,702 samples with an equal class distribution of 33.3% for each class, enabling the classifiers to learn more balanced and discriminative patterns across all fetal health categories.

**Table 2:** Class distribution of the training set before and after applying SMOTEENN.

Training stage	Normal	Suspect	Pathological	Total
Before SMOTEENN	1,234 (77.9%)	219 (13.8%)	131 (8.3%)	1,584
After SMOTEENN	1,234 (33.3%)	1,234 (33.3%)	1,234 (33.3%)	3,702

It should be noted that while SMOTEENN effectively rebalances the dataset, the synthetic samples generated by SMOTE are interpolations in feature space and may not perfectly represent real clinical cases. To avoid data leakage and prevent artificially inflated performance, the dataset was first divided into training and testing subsets using a stratified train–test split. SMOTEENN was then applied only to the training data, while the test set was kept completely untouched to represent unseen clinical cases. The ENN cleaning step further helped remove noisy synthetic and original samples that were misclassified by their nearest neighbors, thereby improving the quality of the resampled training data as discussed in Section 3.4. This approach ensures that model learning benefits from class balancing while final evaluation remains unbiased and clinically realistic.

### 3.5 Feature selection

During feature selection, the Variance Inflation Factor (VIF) was employed to detect and reduce multicollinearity among the independent variables. Multicollinearity occurs when two or more predictors in a model are highly correlated, leading to redundant information and unstable regression coefficients, particularly in linear models such as Logistic Regression. The VIF quantifies the degree to which a regression coefficient is inflated due to correlation with other predictors.

Table 3 presents the VIF values for the 16 candidate input features in the fetal health prediction model. VIF assesses multicollinearity by quantifying how well each predictor can be explained by the remaining predictors. Generally, a VIF value above 5 indicates moderate multicollinearity, while values exceeding 10 suggest severe redundancy.

**Table 3:** Variance Inflation Factor (VIF) values for each feature.

Sr. No.	Feature	VIF
1	Baseline value	5.53
2	Accelerations	2.79
3	Uterine contractions	1.26
4	Light decelerations	3.38
5	Prolonged decelerations	2.37
6	Abnormal short-term variability	1.86
7	Mean value of short-term variability	2.90
8	Percentage of time with abnormal long-term variability	1.86
9	Mean value of long-term variability	2.03
10	Histogram width	∞
11	Histogram min	**∞**
12	Histogram max	**∞**
13	Histogram number of peaks	2.33
14	Histogram mean	**10.74**
15	Histogram variance	2.49
16	Histogram tendency	2.52

Bold values indicate VIF = ∞ (perfect multicollinearity), identifying features removed during feature selection.

In this analysis, Histogram Mean produced the highest finite VIF value of 10.74, indicating strong overlap with other histogram-derived variables. Similarly, Baseline Value (5.53), Light Decelerations (3.38), and Mean Value of Short-Term Variability (2.90) showed comparatively higher VIF scores, suggesting partial redundancy. In addition, Histogram Min, Histogram Max, and Histogram Width returned infinite VIF values (∞), which indicates perfect linear dependence among these histogram features, since Histogram Width is directly related to the difference between maximum and minimum histogram values.

Therefore, features with infinite or excessively high VIF values were treated as redundant and considered for removal during feature selection. This helped reduce multicollinearity, improve model interpretability, and maintain numerical stability, particularly for models such as Logistic Regression and other algorithms that may be affected by highly correlated predictors. Features with low VIF values, such as uterine contractions and abnormal short-term variability, were retained because they contributed relatively independent information to the prediction task.

### 3.6 Statistical analysis

Statistical analysis plays an essential role in healthcare data analysis, particularly for fetal health prediction. Before applying machine learning (ML) and neural network models, the dataset must be examined using appropriate statistical methods to understand feature distributions, class patterns, and relationships among variables. This step ensures that data cleaning, feature selection, and model preparation are evidence-based rather than assumption-driven.

In this study, statistical analysis was employed to examine CTG-based features and assess distributional variations across the three fetal health classes. This step identified features that exhibited statistically significant differences among the Normal, Suspect, and Pathological classes. Based on this analysis, only features demonstrating meaningful class-separation ability were retained for model development, thereby enhancing the reliability and interpretability of the fetal health classification framework.

#### 3.6.1 Kruskal–Wallis H-test

**Figure 11:**
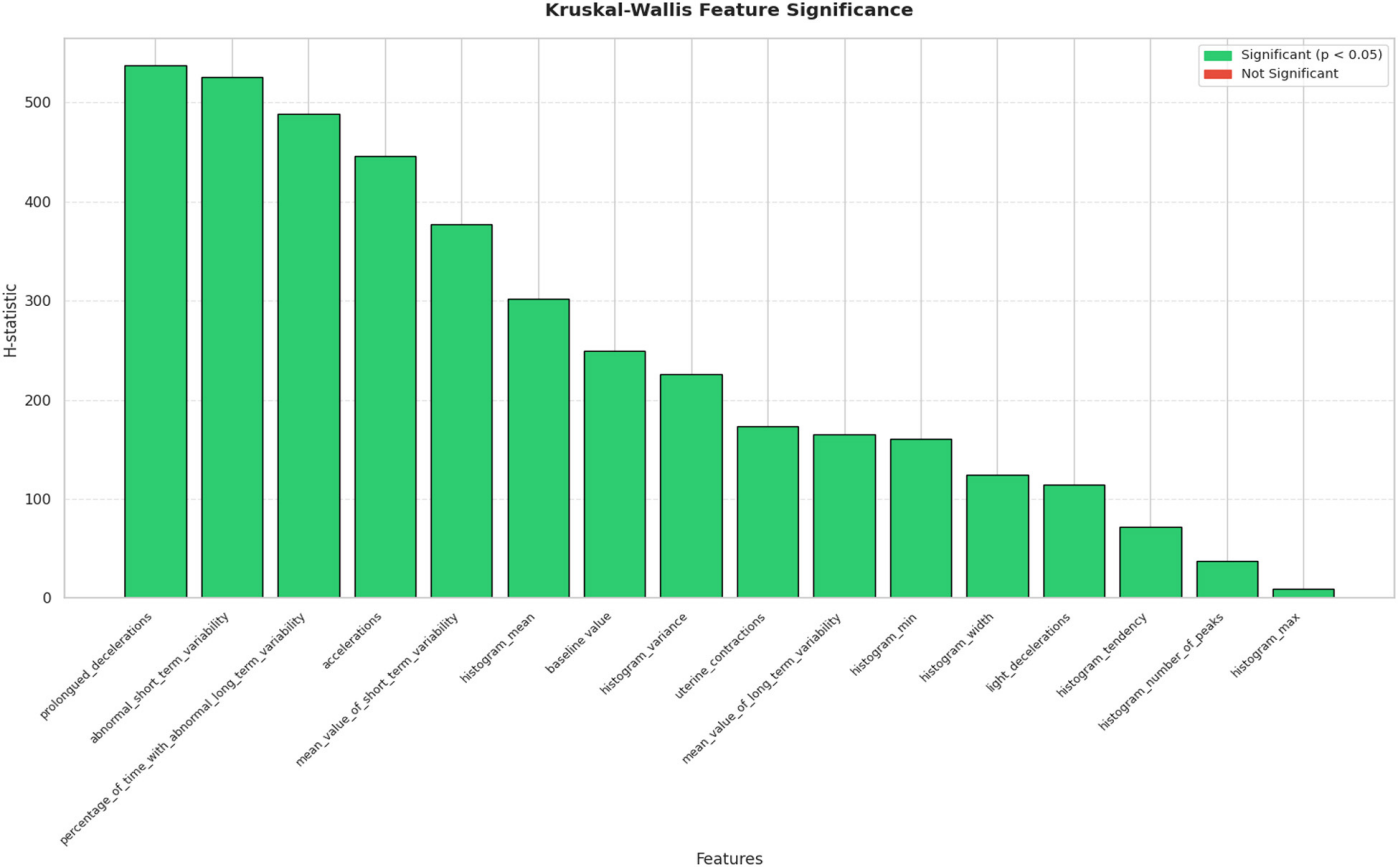
Bar plot showing the results of the Kruskal–Wallis H-test performed on each feature across the three fetal health classes: normal (Class 1), suspect (Class 2), and pathological (Class 3).

After exploratory analysis and correlation-based filtering, the Kruskal–Wallis H-test was used to check whether each numerical feature showed meaningful differences across the three fetal health classes, as shown in Figure 11. Since this test does not assume a normal distribution, it was suitable for the CTG dataset, where several features were skewed and the class sizes were unequal.

This test was suitable for the present dataset because several CTG-derived features were non-normally distributed or skewed, and the class distribution was imbalanced, particularly for the Suspect and Pathological categories. Therefore, the test helped identify features with statistically meaningful differences across fetal health classes without assuming normality.

##### 3.6.1.1 Intuition

The test works by replacing raw feature values with their global *rank* across all observations, then checking whether the average rank differs meaningfully across the three health classes. If a feature has no discriminative power, the rank averages across classes should be roughly equal. A large spread in rank averages captured by the H-statistic suggests the feature separates the classes and should be retained for model training.

##### 3.6.1.2 Ranking procedure

Let the combined, pooled dataset across all *k* = 3 groups contain *N* observations in total. Every observation *x*_*ij*_ (the *j*-th sample in group *i*) is replaced by its rank *r*_*ij*_ when all *N* values are sorted in ascending order. Tied values receive the average of the ranks they would otherwise occupy (mid-rank convention):


$$\begin{array}{l} r_{ij}=\frac{1}{\left|T\right|}\underset{t\in T}{\sum }t, \end{array}$$ (1)

where T is the set of rank positions occupied by the tied values. The rank sum for group *i* is then $R_{i}=\overset{n_{i}}{\underset{j=1}{\sum }}r_{ij}$, and the *mean rank* for that group is ${\bar{R}}_{i}=R_{i}/n_{i}$.

##### 3.6.1.3 H-statistic

The Kruskal–Wallis H-statistic measures the weighted deviation of each group's mean rank from the grand mean rank (*N*+1)/2:


$$\begin{array}{l} H=\frac{12}{N\left(N+1\right)}\overset{k}{\underset{i=1}{\sum }}\frac{R_{i}^{2}}{n_{i}}-3\left(N+1\right), \end{array}$$ (2)

where:

- *N* = total number of observations across all groups (*N* = 2, 126 in our study, or *N* = 3, 702 after SMOTEENN; the test was applied *before* resampling on the original class labels),
- *k* = number of independent groups (*k* = 3: Normal, Suspect, Pathological),
- *n*_*i*_ = number of observations in group *i*, and
- *R*_*i*_ = sum of ranks assigned to observations in group *i*.

Equation 2 can be rewritten in a form that makes the within-group vs. between-group comparison explicit. Define the grand mean rank $\bar{R}=\left(N+1\right)/2$. Then:


$$\begin{array}{l} H=\frac{12}{N\left(N+1\right)}\overset{k}{\underset{i=1}{\sum }}n_{i}{\left({\bar{R}}_{i}-\bar{R}\right)}^{2}, \end{array}$$ (3)

Which is directly analogous to the between group sum of squares in one-way ANOVA, computed on the ranks rather than on the raw values. This formulation makes clear that *H* increases whenever the class-specific mean ranks ${\bar{R}}_{i}$ diverge from the global mean. i.e., whenever the feature tends to take systematically higher or lower values in some health classes than in others.

##### 3.6.1.4 Correction for ties

When *g* groups of tied observations are present, with the ℓ-th tie group containing *t*_ℓ_ identical values, the H-statistic is divided by a correction factor *C* to avoid underestimation:


$$\begin{array}{l} C=1-\frac{\sum_{\ell =1}^{g}\left(t_{\ell }^{3}-t_{\ell }\right)}{N^{3}-N}, \end{array}$$ (4)

Giving the tie-corrected statistic *H*_*c*_ = *H*/*C*. Since CTG features such as histogram_number_of_zeroes contain large numbers of zero values (ties), this correction was applied in our implementation to ensure the p-values are not artificially inflated.

##### 3.6.1.5 Asymptotic distribution and hypothesis test

Under the null hypothesis *H*_0_ that all *k* populations have identical distributions, and provided each group has at least five observations (*n*_*i*_≥5), the statistic *H* (or *H*_*c*_) follows approximately a chi-squared distribution with *k*−1 degrees of freedom:


$$\begin{array}{l} H\sim \chi_{k-1}^{2}\;\text{under }H_{0}. \end{array}$$ (5)

In our case, *k*−1 = 2 degrees of freedom. The hypotheses are:

- Null hypothesis (*H*_0_): The distribution of the feature is identical across all three fetal health classes; equivalently, the population median ranks are equal.
- Alternative hypothesis (*H*_*a*_): At least one fetal health class has a statistically different distribution, indicating that at least one group differs in median rank.

If the computed p-value satisfies *p* < α = 0.05, the null hypothesis (*H*_0_) is rejected, indicating that the corresponding feature shows statistically significant variation across fetal health classes. Such features may provide useful discriminative information for classification. Conversely, features that fail to reject *H*_0_ do not show statistically reliable separation among the classes and may be considered for removal during feature selection.

### 3.7 Model development

After preprocessing, the dataset was divided using a stratified 75:25 train–test split to preserve the original class distribution in both subsets. Nine machine learning (ML) classifiers were trained and compared: Logistic Regression, Decision Tree, SVM, Random Forest, LightGBM, Gradient Boosting, AdaBoost, KNN, and XGBoost. These algorithms were selected based on their proven effectiveness in structured healthcare classification tasks.

Along with the machine learning models, two neural network-based models were also developed for multiclass fetal health prediction. The first was a MLP, shown in Figure 14. A deeper fully connected neural network was also implemented, as shown in Figure 15, to learn more complex non-linear relationships from the selected CTG features.

Hyperparameter tuning was carried out using grid search with cross-validation, as summarized in Table 4. For each model, the main parameters were selected based on their effect on model complexity, generalization, and training efficiency. For example, the learning rate and number of estimators were tuned for ensemble models, the kernel type and regularization parameters were adjusted for SVM, and the hidden layer structure was optimized for the MLP model. The best hyperparameter settings obtained during cross-validation.

**Table 4:** Overview of ML models and hyperparameter configurations.

Sr No	Model	Tuning parameters
1	Logistic regression	multi_class='multinomial', solver='lbfgs', penalty='l2', *C*=10, class_weight='balanced', max_iter=1000
2	K-Nearest Neighbors	*k*=7, weights='distance', metric='manhattan', leaf_size=20
3	SVM (Pipeline)	*C*=50, kernel='rbf', γ='scale', probability=True (with StandardScaler)
4	Random forest	*n*_est_=300, max_depth=15, min_samples_split=2, min_samples_leaf=1, max_features='sqrt', bootstrap=True
5	MLP	hidden_layer_sizes=(128,64), activation='relu', solver='adam', learning_rate='adaptive', η_0_=0.001, max_iter=500, early_stopping=True
6	Decision tree	criterion='entropy', max_depth=12, min_samples_split=5, min_samples_leaf=2, max_features='log2', ccp_alpha=0.001
7	LightGBM	objective='multiclass', boosting_type='gbdt', num_leaves=63, max_depth=8, *n*_est_=500, η=0.05, subsample=0.8, colsample_bytree=0.8, λ_*l*2_=0.1
8	AdaBoost	base_estimator=DecisionTreeClassifier(max_depth=3, criterion='entropy'), *n*_est_=200, learning_rate=0.5
9	Gradient boosting	*n*_est_=200, max_depth=4, η=0.05, subsample=0.8, min_samples_split=5, max_features='sqrt'
10	XGBoost	objective='multi:softprob', *n*_est_=500, max_depth=6, η=0.05, subsample=0.8, colsample_bytree=0.8, λ=1.0, α=0.1, min_child_weight=5

### 3.8 Model evaluation and performance metrics

After training the 11 classifiers described in Section 3.7, their performance was evaluated using multiple complementary metrics. This multi-metric approach was adopted to provide a comprehensive and balanced analysis of model behavior across all fetal health classes. Such an evaluation is essential for fetal health classification because the minority classes, Suspect and Pathological, are clinically critical and may not be adequately reflected by overall accuracy alone.

#### 3.8.1 Confusion-matrix-based evaluation

The confusion matrix was used as the primary basis for deriving classification performance metrics such as accuracy, precision, recall, and F1-score. It summarizes the relationship between the actual and predicted class labels by showing the number of correctly and incorrectly classified samples for each fetal health class. In this study, the three fetal health classes are represented as Class 1, Class 2, and Class 3, corresponding to Normal, Suspect, and Pathological conditions, respectively.

For a multiclass classification problem, the confusion matrix can be represented as shown in Table 5. In this matrix, *m*_*ij*_ denotes the number of samples whose actual class is Class *i* and predicted class is Class *j*. Therefore, rows represent actual classes, while columns represent predicted classes. The diagonal elements represent correctly classified samples, whereas the off-diagonal elements represent misclassified samples.

**Table 5:** Confusion matrix representing predictions across three classes.

	Predicted: Class 1	Predicted: Class 2	Predicted: Class 3
Actual: Class 1	*m*_11_	*m*_12_	*m*_13_
Actual: Class 2	*m*_21_	*m*_22_	*m*_23_
Actual: Class 3	*m*_31_	*m*_32_	*m*_33_

Based on Table 5, the class-wise true positives, false positives, and false negatives were computed for each class. For Class *i*, these values are defined as follows:


$$\begin{array}{l} TP_{i}=m_{ii} \end{array}$$ (6)

$$\begin{array}{l} FP_{i}=\overset{C}{\underset{\begin{array}{c} j=1\\ j\neq i \end{array}}{\sum }}m_{ji} \end{array}$$ (7)

$$\begin{array}{l} FN_{i}=\overset{C}{\underset{\begin{array}{c} j=1\\ j\neq i \end{array}}{\sum }}m_{ij} \end{array}$$ (8)

where *TP*_*i*_ represents the number of correctly classified samples of class *i*, *FP*_*i*_ represents the number of samples from other classes incorrectly predicted as class *i*, and *FN*_*i*_ represents the number of samples of class *i* incorrectly predicted as another class. Here, *C* denotes the total number of classes and *N* denotes the total number of samples.

1. Accuracy
  Accuracy measures the overall proportion of correctly classified samples among all test samples. It is calculated as:
  $$\begin{array}{l} \text{Accuracy}=\frac{\sum_{i=1}^{C}TP_{i}}{N} \end{array}$$ (9)
  where *TP*_*i*_ is the number of true positives for class *i*, *C* is the total number of classes, and *N* is the total number of samples.
2. Precision
  Precision measures the proportion of correctly predicted samples for a given class among all samples predicted as that class. For class *i*, it is calculated as:
  $$\begin{array}{l} {\text{Precision}}_{i}=\frac{TP_{i}}{TP_{i}+FP_{i}} \end{array}$$ (10)
  where *TP*_*i*_ is the number of true positives and *FP*_*i*_ is the number of false positives for class *i*.
3. Recall
  Recall measures the proportion of actual samples of a given class that are correctly identified by the model. For class *i*, it is calculated as:
  $$\begin{array}{l} {\text{Recall}}_{i}=\frac{TP_{i}}{TP_{i}+FN_{i}} \end{array}$$ (11)
  where *FN*_*i*_ is the number of false negatives for class *i*.
4. F1-Score
  The F1-score provides a harmonic mean of precision and recall, making it useful when both false positives and false negatives are important. For class *i*, it is calculated as:
  $$\begin{array}{l} {\text{F1-Score}}_{i}=\frac{2\times {\text{Precision}}_{i}\times {\text{Recall}}_{i}}{{\text{Precision}}_{i}+{\text{Recall}}_{i}} \end{array}$$ (12)

#### 3.8.2 Imbalance-aware evaluation metrics

To assess model robustness beyond overall classification performance, we also employed additional evaluation metrics that are more suitable for imbalanced medical datasets. These metrics provide a fairer assessment of model performance by considering class-wise behavior, particularly for clinically important minority classes.

1. Balanced Accuracy
  Balanced accuracy accounts for per-class performance equally, regardless of class frequency. It is defined as the arithmetic mean of class-wise recall:
  $$\begin{array}{l} \text{Balanced Accuracy}=\frac{1}{C}\overset{C}{\underset{i=1}{\sum }}\frac{TP_{i}}{TP_{i}+FN_{i}} \end{array}$$ (13)
  This metric is particularly useful for fetal health classification because it prevents the majority class from dominating the overall evaluation.
2. Macro-Averaged F1 Score
  The macro-averaged F1 score computes the F1-score independently for each class and averages them without weighting by class support (sample size). This approach penalizes poor performance on minority classes (Suspect and Pathological) and provides a balanced assessment of model performance across all three fetal health categories.
  $$\begin{array}{l} F1_{i}=\frac{2\cdot {\text{Precision}}_{i}\cdot {\text{Recall}}_{i}}{{\text{Precision}}_{i}+{\text{Recall}}_{i}} \end{array}$$ (14)
  $$\begin{array}{l} \text{Macro-}F1=\frac{1}{C}\overset{C}{\underset{i=1}{\sum }}F1_{i} \end{array}$$ (15)
3. Matthews Correlation Coefficient (MCC)
  The Matthews Correlation Coefficient provides a single scalar measure that captures the overall quality of classification using the complete confusion matrix. It remains informative even under class imbalance and is therefore suitable for medical classification tasks (Chicco and Jurman, 2020). For the multiclass setting with *C* classes, MCC is computed from the *C*×*C* confusion matrix **M**, where *m*_*ij*_ denotes the number of samples of true class *i* predicted as class *j* (Gorodkin, 2004):
  $$\begin{array}{l} \text{MCC}=\frac{N\overset{C}{\underset{k=1}{\sum }}m_{kk}-\overset{C}{\underset{k=1}{\sum }}t_{k}p_{k}}{\sqrt{\left(N^{2}-\overset{C}{\underset{k=1}{\sum }}p_{k}^{2}\right)\left(N^{2}-\overset{C}{\underset{k=1}{\sum }}t_{k}^{2}\right)}} \end{array}$$ (16)
  where *N* represents the total number of samples, $t_{k}=\underset{j}{\sum }m_{kj}$ denotes the total number of samples belonging to the true class *k*, and $p_{k}=\underset{i}{\sum }m_{ik}$ represents the total number of samples predicted as class *k*. The MCC value ranges from −1 to +1, where +1 indicates perfect classification, 0 represents performance equivalent to random prediction, and −1 indicates complete disagreement between the predicted and true class labels.
4. Cohen's Kappa (κ)
  Cohen's Kappa measures the level of agreement between the predicted and ground-truth class labels while accounting for the agreement that may occur by chance (Cohen, 1960). It is computed as:
  $$\begin{array}{l} \kappa =\frac{p_{o}-p_{e}}{1-p_{e}} \end{array}$$ (17)
  where *p*_*o*_ denotes the observed agreement between the predicted and true class labels, which is equivalent to the overall accuracy, and *p*_*e*_ represents the expected agreement occurring by chance:
  $$\begin{array}{l} p_{e}=\overset{C}{\underset{i=1}{\sum }}\frac{{\text{actual}}_{i}\times {\text{predicted}}_{i}}{N^{2}} \end{array}$$ (18)
  where *N* is the total number of samples. A value of κ = 1 indicates perfect agreement, κ = 0 indicates chance-level agreement, and κ < 0 indicates systematic disagreement.

Together, these metrics provide a statistically robust and imbalance-aware evaluation framework, enabling a fair and clinically meaningful comparison of model performance across all experimental settings.

#### 3.8.3 ROC curve and AUC analysis

The Receiver Operating Characteristic (ROC) curve and Area Under the Curve (AUC) were used to assess the discriminative ability of each model in separating the fetal health classes, as shown in Figure 17. The ROC curve plots the True Positive Rate (TPR, sensitivity) against the False Positive Rate (FPR) at various classification thresholds, illustrating the trade-off between correctly detecting a class and minimizing false positive predictions.

Since fetal health prediction involves three classes, the ROC curves were generated using a one-vs-rest (OvR) approach. In this method, one class is treated as the positive class, and the remaining two classes are grouped as negative. The AUC gives a single score for class separability, where a higher value indicates better discrimination between fetal health categories.

#### 3.8.4 Cross-validation

To evaluate the performance of the proposed machine learning and neural network-based models, 10-fold cross-validation was used in this study. This approach provides a more reliable estimate of model generalization by testing model performance across different training and validation splits. It also helps reduce the risk of overfitting, which is important in fetal health classification because the dataset is imbalanced and the minority classes, Suspect and Pathological, require careful evaluation.

In 10-fold cross-validation, the training data were divided into 10 approximately equal stratified folds to preserve the class distribution in each fold. In each round, one fold was used for validation, while the remaining nine folds were used for training. This process was repeated 10 times, with each fold used once as the validation set. The final cross-validation result was reported as the average performance across all folds, giving a more stable estimate of model performance.

### 3.9 Web application development

**Figure 12:**
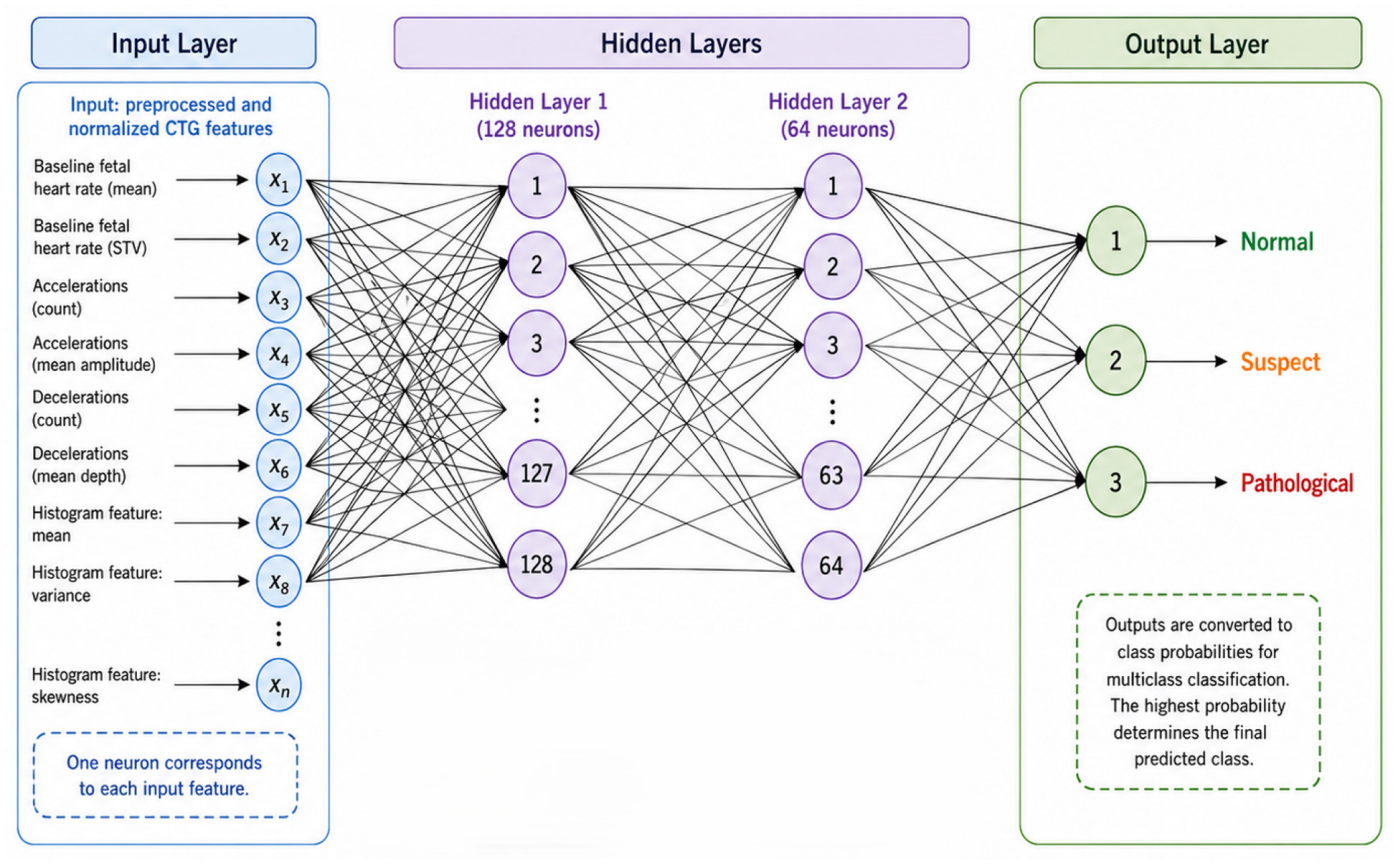
Multi-layer perceptron architecture.

**Figure 13:**
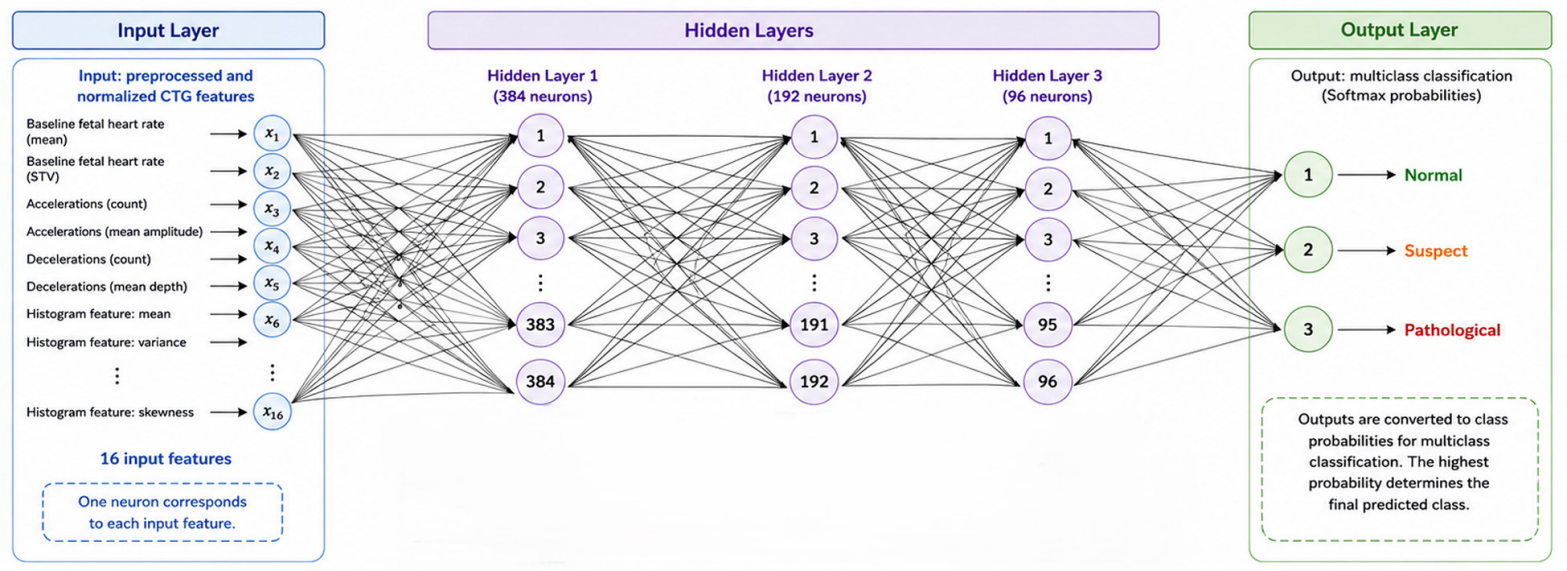
Architecture of the DNN model.

A web-based application was developed to make the trained model accessible and user-friendly. The application was built using the Streamlit framework, as illustrated in Figures 12, 13. The interface enables healthcare professionals, technicians, and community health workers to input clinical features from cardiotocographic (CTG) recordings and obtain real-time predictions of fetal health status. The application accepts key CTG-derived attributes as input, including baseline fetal heart rate, accelerations, decelerations, uterine contractions, and variability indices. Upon submission, the input data is processed by the backend using the trained LightGBM model described in Section 3.7, which returns the predicted fetal health class.

Figure 13 followed a step-by-step approach to develop a fetal health prediction system.

Step 1: The user provides input data through an intuitive web interface built using Streamlit.

Step 2: The input data is transmitted to the backend via a request mechanism for further processing.

Step 3: The backend executes the machine learning logic to load the appropriate settings and pre-trained LightGBM model. The model then performs real-time analysis and classification of the fetal condition.

Step 4: The backend returns the prediction result categorized as Normal, Suspect, or Pathological back to the frontend.

Step 5: The result is displayed clearly to the user through the Streamlit interface.

The technology stack used for developing and deploying the web application is summarized in Table 6. The selected tools support interactive user interface development, model loading, data preprocessing, and lightweight deployment of the proposed fetal health prediction system.

**Table 6:** Technology stack used for experimentation results.

Library	Purpose
Streamlit	Used as the frontend framework for developing the interactive web application with real-time rendering and user input widgets.
Scikit-learn	Used for loading and executing the pre-trained LightGBM model for fetal health prediction.
Pandas	Used for data manipulation, input handling, and preprocessing before passing the data to the trained model.
NumPy	Used for numerical operations and array-based data processing required during model inference.
Pickle	Used for model serialization and deserialization, allowing the trained model to be saved and loaded within the web application.
Python Script	Used to run the entire application as a single-page lightweight program, making it easy to deploy on cloud platforms or local servers without requiring a separate backend framework.

## 4 Applied machine learning and neural network-based techniques

In this study, nine conventional machine learning algorithms and two neural network-based models were used for fetal health classification. Each model follows a different learning approach to map CTG-derived features to the corresponding fetal health class. This section briefly describes the applied models and their main mathematical formulations.

### 4.1 Logistic regression

Multiclass Logistic Regression extends binary logistic regression to handle more than two classes by using the softmax function. The softmax function converts the model outputs into class probabilities, ensuring that the probabilities across all classes sum to one. This makes Logistic Regression useful for directly estimating the probability of each fetal health class (LaValley, 2008).

For a given input feature vector **x**, the model computes a score, also called a logit, for each class *i* as:


$$\begin{array}{l} z_{i}={\text{w}}_{i}^{T}\text{x}+b_{i} \end{array}$$ (19)

where **w**_*i*_ represents the weight vector for class *i*, *b*_*i*_ is the bias term, and *z*_*i*_ is the computed logit score. These scores are then transformed into normalized class probabilities using the softmax function:


$$\begin{array}{l} P\left(y=i|\text{x}\right)=\frac{e^{z_{i}}}{\sum_{j=1}^{K}e^{z_{j}}} \end{array}$$ (20)

where *K* denotes the total number of fetal health classes. The final predicted class is assigned by selecting the class with the highest probability:


$$\begin{array}{l} ŷ=\arg\underset{i}{\max}P\left(y=i|\text{x}\right) \end{array}$$ (21)

This probabilistic interpretation makes logistic regression interpretable and suitable for scenarios requiring confidence scores. The model is trained by minimizing the categorical cross-entropy loss function:


$$\begin{array}{l} L=-\overset{K}{\underset{i=1}{\sum }}y_{i}\log\left(P\left(y=i|\text{x}\right)\right) \end{array}$$ (22)

often with L2 regularization to avoid overfitting. Even though logistic regression is linear in nature, it can perform surprisingly well when the class boundaries are approximately linear in the input space.

### 4.2 K-Nearest Neighbors (KNN)

One of the simplest and most effective supervised learning algorithms used for classification is the KNN algorithm. K Nearest Neighbors is a type of non-parametric, instance-based learning algorithm that does not make any assumptions about the underlying distribution of the dataset. It actually stores the training dataset and predicts (Guo et al., 2003).

K-Nearest Neighbors works by searching for the *k* nearest neighbors of a new instance, via some distance metric, most commonly the Euclidean distance, and classifying this new instance according to majority voting among them. The Euclidean distance between two data points **x** and **x**_*i*_ is computed as:


$$\begin{array}{l} d\left(\text{x},{\text{x}}_{i}\right)=\sqrt{\overset{n}{\underset{j=1}{\sum }}{\left(x_{j}-x_{i,j}\right)}^{2}} \end{array}$$ (23)

where *n* is the number of features present in the dataset. For multi-class classification with *C* classes {*c*_1_, *c*_2_, …, *c*_*C*_}, the algorithm selects the *k* samples with the smallest distances and assigns the class with the highest number of votes among them to the new instance:


$$\begin{array}{l} ŷ=\arg\underset{c\in \left\{c_{1},\ldots ,c_{C}\right\}}{\max}\overset{k}{\underset{i=1}{\sum }}1\left[y_{i}=c\right] \end{array}$$ (24)

where **1**[·] is the indicator function and *y*_*i*_ is the class label of the *i*-th nearest neighbor.

### 4.3 Support Vector Machine (SVM)

Support Vector Machines (SVMs) are large-margin classifiers that aim to find the hyperplane that best separates data points from different classes. In the multiclass setting, the common strategy is *One-vs-Rest (OvR)*, where *K* binary classifiers are trained (Mathur and Foody, 2008). The optimization problem for each binary classifier is:


$$\begin{array}{l} \underset{\text{w},b}{\min}\;\frac{1}{2}∥\text{w}{∥}^{2}+C\overset{n}{\underset{i=1}{\sum }}\max\left(0,1-y_{i}\left({\text{w}}^{T}{\text{x}}_{i}+b\right)\right) \end{array}$$ (25)

where *C* is the regularization parameter, **w** is the weight vector, *b* is the bias term, and *y*_*i*_∈{−1, +1} is the binary class label for each OvR classifier. During inference, the class with the highest decision function value is selected:


$$\begin{array}{l} ŷ=\arg\underset{k\in \left\{1,\ldots ,K\right\}}{\max}\left({\text{w}}_{k}^{T}\text{x}+b_{k}\right) \end{array}$$ (26)

where *K* is the total number of classes, **w**_*k*_ and *b*_*k*_ are the weight vector and bias of the *k*-th classifier respectively. An alternative strategy is *One-vs-One (OvO)*, where $\frac{K\left(K-1\right)}{2}$ classifiers are trained for all possible class pairs. The final prediction is made using majority voting:


$$\begin{array}{l} ŷ=\arg\underset{k\in \left\{1,\ldots ,K\right\}}{\max}\underset{l\neq k}{\sum }1\left[f_{kl}\left(\text{x}\right)>0\right] \end{array}$$ (27)

where *f*_*kl*_(**x**) is the decision function of the binary classifier trained between classes *k* and *l*, and **1**[·] is the indicator function. SVMs are effective in high-dimensional spaces and with small datasets.

### 4.4 Decision trees

Decision Trees are non-parametric algorithms that recursively partition the feature space into subsets to improve class purity (Ying, 2015). At each internal node, the best split is chosen by selecting the feature *j* and threshold *t* that maximizes the Information Gain:


$$\begin{array}{l} \text{IG}\left(S,j,t\right)=\text{H}\left(S\right)-\frac{\left|S_{L}\right|}{\left|S\right|}\text{H}\left(S_{L}\right)-\frac{\left|S_{R}\right|}{\left|S\right|}\text{H}\left(S_{R}\right) \end{array}$$ (28)

where *S*_*L*_ = {**x**_*i*_∈*S*∣*x*_*ij*_ ≤ *t*} and *S*_*R*_ = {**x**_*i*_∈*S*∣*x*_*ij*_>*t*} are the left and right subsets after the split. The quality of each subset is evaluated using either Gini Impurity or Entropy:

Gini Impurity:


$$\begin{array}{l} G\left(S\right)=1-\overset{K}{\underset{k=1}{\sum }}p_{k}^{2} \end{array}$$ (29)

Entropy:


$$\begin{array}{l} H\left(S\right)=-\overset{K}{\underset{k=1}{\sum }}p_{k}\log_{2}\left(p_{k}\right) \end{array}$$ (30)

where *K* is the total number of classes and *p*_*k*_ is the proportion of instances belonging to class *k* at a given node. The tree construction continues until a stopping criterion is met, such as maximum depth or minimum samples per leaf. For multi-class classification, the predicted class at each leaf node is:


$$\begin{array}{l} ŷ=\arg\underset{k\in \left\{1,\ldots ,K\right\}}{\max}\underset{i\in S_{\text{leaf}}}{\sum }1\left[y_{i}=k\right] \end{array}$$ (31)

where *S*_leaf_ is the set of training instances that reach that leaf. While simple and interpretable, decision trees are prone to overfitting.

### 4.5 Random forest

Random Forest is an ensemble of decision trees, where each tree is trained on a bootstrap sample of the data and uses a random subset of features at each split (Speiser et al., 2019). For multi-class classification with *K* classes, each tree *h*_*t*_(**x**) produces a prediction, and the final class is determined by majority voting across all *T* trees:


$$\begin{array}{l} ŷ=\arg\underset{k\in \left\{1,\ldots ,K\right\}}{\max}\overset{T}{\underset{t=1}{\sum }}1\left[h_{t}\left(\text{x}\right)=k\right] \end{array}$$ (32)

where *T* is the number of trees, *h*_*t*_(**x**) is the prediction of the *t*-th tree, and **1**[·] is the indicator function. The class probability estimate for class *k* can also be computed as:


$$\begin{array}{l} \hat{P}\left(y=k|\text{x}\right)=\frac{1}{T}\overset{T}{\underset{t=1}{\sum }}1\left[h_{t}\left(\text{x}\right)=k\right] \end{array}$$ (33)

Bootstrap sampling introduces diversity among the individual trees, while random feature selection at each split reduces the correlation between them. This combination helps lower model variance and improves generalization, making Random Forest a robust and effective algorithm for multiclass classification problems.

### 4.6 Gradient boosting

Gradient Boosting is an ensemble learning technique that builds a strong predictive model by sequentially combining multiple weak learners, usually decision trees. Unlike Random Forest, where trees are trained independently, Gradient Boosting trains each new learner to correct the errors made by the previous learners. This sequential learning process enables the model to gradually improve its predictive performance by minimizing a differentiable loss function (Friedman, 2001).

For a given input vector **x**, the model prediction at iteration *m* is updated as:


$$\begin{array}{l} F^{\left(m\right)}\left(\text{x}\right)=F^{\left(m-1\right)}\left(\text{x}\right)+\eta \cdot h_{m}\left(\text{x}\right) \end{array}$$ (34)

where *F*^(*m*)^(**x**) represents the model prediction after the *m*-th iteration, *F*^(*m*−1)^(**x**) is the prediction from the previous iteration, η is the learning rate, and *h*_*m*_(**x**) is the weak learner added at iteration *m*.

For multi-class classification with *K* classes, Gradient Boosting maintains class-wise prediction functions. The predicted probability for class *k* is obtained using the softmax function:


$$\begin{array}{l} p_{ik}=\frac{\exp\left(F_{k}\left({\text{x}}_{i}\right)\right)}{\sum_{k^{\prime }=1}^{K}\exp\left(F_{k^{\prime }}\left({\text{x}}_{i}\right)\right)} \end{array}$$ (35)

where *F*_*k*_(**x**_*i*_) is the raw prediction score for class *k*, and *p*_*ik*_ is the predicted probability that sample **x**_*i*_ belongs to class *k*.

The model is trained by minimizing the multi-class cross-entropy loss function:


$$\begin{array}{l} L=-\overset{N}{\underset{i=1}{\sum }}\overset{K}{\underset{k=1}{\sum }}y_{ik}\log\left(p_{ik}\right) \end{array}$$ (36)

where *N* denotes the total number of training samples, *K* represents the number of fetal health classes, *y*_*ik*_ is the one-hot encoded true label for sample *i* and class *k*, and *p*_*ik*_ is the predicted probability assigned to class *k*.

At each boosting iteration, a weak learner is fitted to the negative gradient of the loss function. For multiclass cross-entropy loss, the pseudo-residual for sample *i* and class *k* can be written as:


$$\begin{array}{l} r_{ik}^{\left(m\right)}=y_{ik}-p_{ik}^{\left(m-1\right)} \end{array}$$ (37)

where $r_{ik}^{\left(m\right)}$ denotes the pseudo-residual at iteration *m*, *y*_*ik*_ represents the actual class indicator, and $p_{ik}^{\left(m-1\right)}$ is the predicted probability from the previous boosting iteration.

After all boosting iterations are completed, the final class label is assigned by selecting the class with the highest predicted probability:


$$\begin{array}{l} ŷ*i=\arg\max*k\in 1,\ldots ,Kp_{ik} \end{array}$$ (38)

Gradient Boosting is effective in capturing complex non-linear relationships and can provide strong predictive performance in multiclass classification tasks. However, because the model is built sequentially, careful tuning of hyperparameters such as the learning rate, number of estimators, and tree depth is required to control overfitting and improve generalization.

### 4.7 Extreme gradient boosting

Extreme Gradient Boosting (XGBoost) builds an ensemble by sequentially adding weak learners that focus on correcting the residual errors of the existing model (Li et al., 2007). For multi-class classification with *K* classes, a separate boosting model is maintained for each class. At iteration *m*, a new weak learner is fitted to the negative gradient of the loss function:


$$\begin{array}{l} F_{k}^{\left(m\right)}\left(\text{x}\right)=F_{k}^{\left(m-1\right)}\left(\text{x}\right)+\eta \cdot h_{k}^{\left(m\right)}\left(\text{x}\right) \end{array}$$ (39)

where $F_{k}^{\left(m\right)}\left(\text{x}\right)$ is the raw score for class *k* at iteration *m*, η is the learning rate, and $h_{k}^{\left(m\right)}\left(\text{x}\right)$ is the new weak learner fitted to the negative gradient of the multi-class cross-entropy loss:


$$\begin{array}{l} r_{i,k}^{\left(m\right)}=-\left[\frac{\partial L\left(y_{i},F^{\left(m-1\right)}\left({\text{x}}_{i}\right)\right)}{\partial F_{k}^{\left(m-1\right)}\left({\text{x}}_{i}\right)}\right] \end{array}$$ (40)

where L is the cross-entropy loss and $r_{i,k}^{\left(m\right)}$ is the pseudo-residual for instance *i* and class *k*. After all iterations, the raw scores are passed through the softmax function to obtain class probabilities:


$$\begin{array}{l} \hat{P}\left(y=k|\text{x}\right)=\frac{\exp\left(F_{k}^{\left(M\right)}\left(\text{x}\right)\right)}{\sum_{k^{\prime }=1}^{K}\exp\left(F_{k^{\prime }}^{\left(M\right)}\left(\text{x}\right)\right)} \end{array}$$ (41)

The final predicted class is then:


$$\begin{array}{l} ŷ=\arg\underset{k\in \left\{1,\ldots ,K\right\}}{\max}\hat{P}\left(y=k|\text{x}\right) \end{array}$$ (42)

XGBoost offers high predictive performance but requires careful tuning to prevent overfitting.

### 4.8 LightGBM

LightGBM (Light Gradient Boosting Machine) is a gradient boosting framework optimized for efficiency and scalability. It employs histogram-based feature binning to speed up computation and grows trees using a leaf-wise strategy (Gu et al., 2023). Similar to XGBoost, a separate boosting model is maintained for each of the *K* classes. The multi-class cross-entropy loss function is:


$$\begin{array}{l} L=-\overset{N}{\underset{i=1}{\sum }}\overset{K}{\underset{k=1}{\sum }}y_{ik}\log\left(p_{ik}\right)+\Omega \left(f\right) \end{array}$$ (43)

where *N* is the number of training instances, *y*_*ik*_∈{0, 1} is the one-hot encoded true label for instance *i* and class *k*, *p*_*ik*_ is the predicted probability, and Ω(*f*) is a regularization term controlling model complexity. The predicted probability for class *k* is obtained via the softmax function:


$$\begin{array}{l} p_{ik}=\frac{\exp\left(F_{k}\left({\text{x}}_{i}\right)\right)}{\sum_{k^{\prime }=1}^{K}\exp\left(F_{k^{\prime }}\left({\text{x}}_{i}\right)\right)} \end{array}$$ (44)

where *F*_*k*_(**x**_*i*_) is the raw score of the *k*-th model for instance *i*. The final predicted class is:


$$\begin{array}{l} {ŷ}_{i}=\arg\underset{k\in \left\{1,\ldots ,K\right\}}{\max}p_{ik} \end{array}$$ (45)

LightGBM's leaf-wise tree growth selects the leaf with the maximum loss reduction at each step, leading to deeper, more accurate trees compared to level-wise strategies. This makes LightGBM particularly effective for large datasets and high-dimensional features.

### 4.9 AdaBoost

AdaBoost (Adaptive Boosting) constructs a strong classifier by combining several weak classifiers in a sequential manner. For multi-class classification, the SAMME (Stagewise Additive Modeling using a Multiclass Exponential loss function) algorithm is applied (Wang and Sun, 2021). At each iteration *m*, a weak learner *h*_*m*_(**x**) is trained on the weighted training set, and its weight is computed based on its weighted error rate:


$$\begin{array}{l} \varepsilon_{m}=\frac{\sum_{i=1}^{N}w_{i}^{\left(m\right)}\cdot 1\left[h_{m}\left({\text{x}}_{i}\right)\neq y_{i}\right]}{\sum_{i=1}^{N}w_{i}^{\left(m\right)}} \end{array}$$ (46)

where $w_{i}^{\left(m\right)}$ is the weight of instance *i* at iteration *m*. The classifier weight is then:


$$\begin{array}{l} \alpha_{m}=\eta \left(\log\left(\frac{1-\varepsilon_{m}}{\varepsilon_{m}}\right)+\log\left(K-1\right)\right) \end{array}$$ (47)

where ε_*m*_ is the weighted error of the *m*-th classifier, *K* is the number of classes, and η is the learning rate. After each iteration, the instance weights are updated to emphasize misclassified samples:


$$\begin{array}{l} w_{i}^{\left(m+1\right)}=w_{i}^{\left(m\right)}\cdot \exp\left(\alpha_{m}\cdot 1\left[h_{m}\left({\text{x}}_{i}\right)\neq y_{i}\right]\right) \end{array}$$ (48)

followed by renormalization so that $\overset{N}{\underset{i=1}{\sum }}w_{i}^{\left(m+1\right)}=1$. The final multi-class prediction is made by a weighted majority vote:


$$\begin{array}{l} ŷ=\arg\underset{k\in \left\{1,\ldots ,K\right\}}{\max}\overset{M}{\underset{m=1}{\sum }}\alpha_{m}\cdot 1\left[h_{m}\left(\text{x}\right)=k\right] \end{array}$$ (49)

where *M* is the total number of weak learners. AdaBoost is sensitive to noisy data but works well with weak learners that perform slightly better than random guessing.

### 4.10 Multi-Layer Perceptron (MLP)

**Figure 14:**
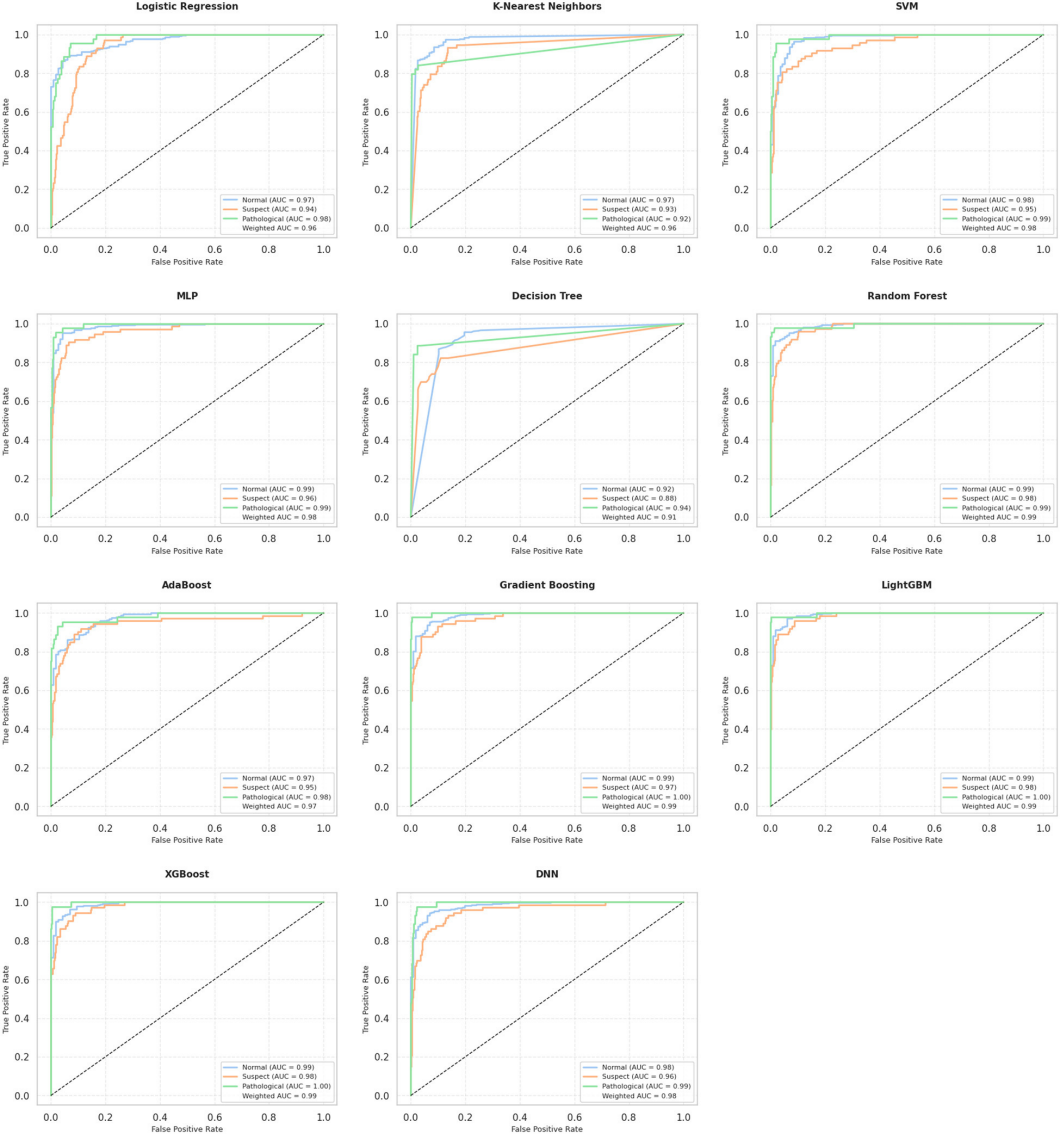
ROC curve comparison of models for fetal health classification.

The Multi-Layer Perceptron is a class of feedforward artificial neural networks widely used for both classification and regression tasks (Tolstikhin et al., 2021). It consists of an input layer, two hidden layers, and an output layer, as shown in Figure 14. Each neuron in a layer is fully connected to every neuron in the subsequent layer. Learning is performed using the backpropagation algorithm, which updates the network weights to minimize the prediction error.

Multi-Layer Perceptrons can model complex, non-linear relationships between input features and target labels. Each neuron computes a weighted sum of its inputs and passes it through a non-linear activation function such as ReLU or sigmoid. Mathematically, the pre-activation *z*^(*l*)^ and output activation *a*^(*l*)^ of layer *l* are given by:


$$\begin{array}{l} z^{\left(l\right)}=W^{\left(l\right)}a^{\left(l-1\right)}+b^{\left(l\right)} \end{array}$$ (50)

$$\begin{array}{l} a^{\left(l\right)}=\sigma \left(z^{\left(l\right)}\right) \end{array}$$ (51)

where $W^{\left(l\right)}\in {\mathbb{R}}^{d_{l}\times d_{l-1}}$ is the weight matrix for layer *l*, $b^{\left(l\right)}\in {\mathbb{R}}^{d_{l}}$ is the bias vector, *a*^(*l*−1)^ is the activation from the previous layer, and σ(·) is a non-linear activation function.

For multi-class classification with *K* classes, the final output layer applies the softmax activation function to produce class probabilities:


$$\begin{array}{l} \hat{P}\left(y=k|\text{x}\right)=\frac{\exp\left(z_{k}^{\left(L\right)}\right)}{\sum_{k^{\prime }=1}^{K}\exp\left(z_{k^{\prime }}^{\left(L\right)}\right)},\;k=1,\ldots ,K \end{array}$$ (52)

where $z_{k}^{\left(L\right)}$ is the *k*-th element of the output pre-activation vector at the final layer *L*, and *K* is the total number of classes. The network is trained by minimizing the categorical cross-entropy loss:


$$\begin{array}{l} L=-\overset{N}{\underset{i=1}{\sum }}\overset{K}{\underset{k=1}{\sum }}y_{ik}\log\left(\hat{P}\left(y=k|{\text{x}}_{i}\right)\right) \end{array}$$ (53)

where *N* is the number of training instances and *y*_*ik*_∈{0, 1} is the one-hot encoded true label for instance *i* and class *k*. The final predicted class is:


$$\begin{array}{l} ŷ=\arg\underset{k\in \left\{1,\ldots ,K\right\}}{\max}\hat{P}\left(y=k|\text{x}\right) \end{array}$$ (54)

Model parameters are optimized using the Adam optimizer, which maintains adaptive learning rates for each parameter using estimates of the first and second moments of the gradients, thereby accelerating convergence. The parameter update rule is:


$$\begin{array}{l} W^{\left(l\right)}\leftarrow W^{\left(l\right)}-\eta \cdot \frac{{\hat{m}}_{t}}{\sqrt{{\hat{v}}_{t}}+\epsilon } \end{array}$$ (55)

where ${\hat{m}}_{t}$ and ${\hat{v}}_{t}$ denote the bias-corrected first and second moment estimates of the gradient, respectively, η represents the learning rate, and ϵ is a small constant added to ensure numerical stability. To reduce overfitting and improve generalization, regularization techniques such as dropout, early stopping, and ℓ_2_ regularization were applied during model training.

#### 4.10.1 MLP framework

The Multi-Layer Perceptron model used in this study includes an input layer, two hidden layer, and an output layer. This structure was chosen because it can learn non-linear relationships from structured CTG-based fetal health features. The main hyperparameters of the MLP model, such as hidden layer size, activation function, optimizer, learning rate, maximum iterations, and early stopping criteria, are summarized in Table 4.

Input Layer: The input layer receives the preprocessed and normalized CTG-based features. These include baseline fetal heart rate, accelerations, decelerations, short-term and long-term variability measures, and histogram-based statistical features. Each selected input feature is represented as an individual input node, allowing the network to process the complete feature set during training. The use of normalized inputs helps improve convergence and prevents features with larger numerical ranges from dominating the learning process.

Hidden Layers: The MLP model employs two hidden layers with 128 and 64 neurons respectively, defined as hidden_layer_sizes = (128,64). Both hidden layers apply the ReLU (Rectified Linear Unit) activation function, which introduces non-linearity and enables the model to learn complex non-linear feature interactions. The Adam optimizer was used for weight updates because it combines adaptive learning rates with momentum-based optimization, improving convergence during training. An adaptive learning rate strategy was applied with an initial learning rate of 0.001. The model was trained with a maximum of 500 iterations, and early stopping was used to terminate training when validation performance no longer improved.

Output Layer: The output layer represents the three fetal health classes: Normal, Suspect, and Pathological. For each input sample, the model produces probability scores for all three classes, and the class with the highest probability is selected as the final prediction. The model was trained using cross-entropy loss, which compares the predicted class probabilities with the actual class label.

### 4.11 Deep Neural Network (DNN)

**Figure 15:**
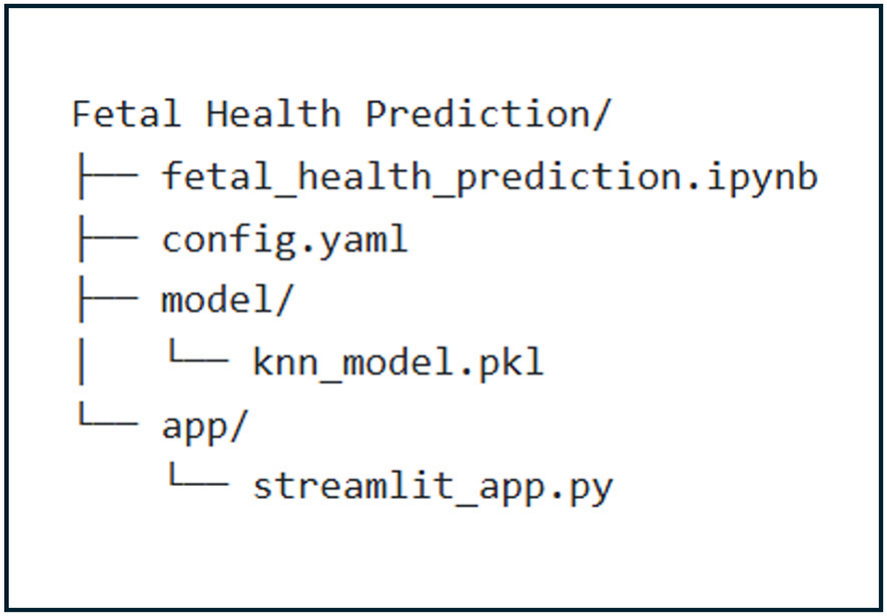
Directory structure of the proposed fetal health prediction system.

The Deep Neural Network used in this study is a fully connected feedforward model developed for multiclass fetal health classification using CTG-based input features. Compared with shallow machine learning models, the DNN uses multiple hidden layers, allowing it to learn more complex non-linear patterns from the selected features. As shown in Figure 15, the proposed architecture consists of an input layer, three fully connected hidden layers, and an output layer for predicting the fetal health class.

In this model, each hidden layer receives the output from the previous layer and applies a dense linear transformation followed by batch normalization, a non-linear activation function, and dropout-based regularization. The dense transformation for the *l*-th hidden layer is mathematically expressed as:


$$\begin{array}{l} u^{\left(l\right)}=W^{\left(l\right)}a^{\left(l-1\right)} \end{array}$$ (56)

where *W*^(*l*)^ represents the weight matrix of layer *l*, and *a*^(*l*−1)^ denotes the activation output from the previous layer. Since batch normalization is applied after each dense layer, the normalized representation can be written as:


$$\begin{array}{l} {ũ}^{\left(l\right)}=\gamma^{\left(l\right)}\frac{u^{\left(l\right)}-\mu_{B}^{\left(l\right)}}{\sqrt{{\left(\sigma_{B}^{\left(l\right)}\right)}^{2}+\epsilon }}+\beta^{\left(l\right)} \end{array}$$ (57)

where $\mu_{B}^{\left(l\right)}$ and ${\left(\sigma_{B}^{\left(l\right)}\right)}^{2}$ denote the batch mean and variance, respectively, while γ^(*l*)^ and β^(*l*)^ are learnable scaling and shifting parameters. The normalized output is then passed through the ReLU activation function:


$$\begin{array}{l} a^{\left(l\right)}=\text{ReLU}\left({ũ}^{\left(l\right)}\right)=\max\left(0,{ũ}^{\left(l\right)}\right) \end{array}$$ (58)

The use of ReLU helps the network learn non-linear patterns from CTG features while reducing the vanishing-gradient problem during training. To improve generalization and reduce overfitting, dropout is applied after each hidden layer. Dropout randomly deactivates a fraction of neurons during training and can be represented as:


$$\begin{array}{l} {ā}^{\left(l\right)}=\frac{r^{\left(l\right)}⊙a^{\left(l\right)}}{1-p_{l}} \end{array}$$ (59)

where *r*^(*l*)^ is a Bernoulli random mask, *p*_*l*_ is the dropout rate of layer *l*, and ⊙ denotes element-wise multiplication.

For the final classification task, the output layer contains three neurons corresponding to the three fetal health classes: Normal, Suspect, and Pathological. The softmax activation function is used to convert the output scores into class probabilities:


$$\begin{array}{l} \hat{P}\left(y=k|\text{x}\right)=\frac{\exp\left(z_{k}^{\left(L\right)}\right)}{\sum_{k^{\prime }=1}^{K}\exp\left(z_{k^{\prime }}^{\left(L\right)}\right)},\;k=1,2,\ldots ,K \end{array}$$ (60)

where *K* = 3 represents the total number of fetal health classes, and $z_{k}^{\left(L\right)}$ is the output score for class *k* at the final layer. The predicted class is obtained by selecting the class with the highest probability:


$$\begin{array}{l} ŷ=\arg\underset{k\in 1,\ldots ,K}{\max}\hat{P}\left(y=k|\text{x}\right) \end{array}$$ (61)

The model is trained using categorical cross-entropy loss with label smoothing, which improves generalization by preventing the model from becoming overconfident in its predictions. The loss function is given by:


$$\begin{array}{l} L=-\overset{N}{\underset{i=1}{\sum }}\overset{K}{\underset{k=1}{\sum }}{ỹ}_{ik}\log\left(\hat{P}\left(y=k|{\text{x}}_{i}\right)\right) \end{array}$$ (62)

where *N* is the number of training samples, *K* is the number of classes, and ỹ_*ik*_ represents the smoothed one-hot encoded label for instance *i* and class *k*. The Adam optimizer is used for parameter optimization due to its adaptive learning-rate mechanism and efficient convergence behavior. The update rule can be expressed as:


$$\begin{array}{l} \theta_{t}\leftarrow \theta_{t-1}-\eta \cdot \frac{{\hat{m}}_{t}}{\sqrt{{\hat{v}}_{t}}+\epsilon } \end{array}$$ (63)

where θ_*t*_ denotes the model parameters at iteration *t*, η is the learning rate, and ${\hat{m}}_{t}$ and ${\hat{v}}_{t}$ are the bias-corrected first and second moment estimates of the gradient, respectively. In addition, early stopping, learning-rate reduction, dropout, batch normalization, and ℓ_2_ regularization are applied to improve training stability and prevent overfitting.

The DNN was implemented as a fully connected feedforward neural network with three hidden layers arranged in a progressively decreasing structure. Each hidden layer used the ReLU activation function to learn non-linear feature representations, while the final output layer used the Softmax activation function to estimate probabilities for the three fetal health classes.

To reduce overfitting on the relatively compact CTG dataset, a multi-component regularization strategy was applied, including batch normalization, dropout, ℓ_2_ weight regularization, and early stopping. The model was trained using the Adam optimizer, and the final hyperparameter settings were selected through a structured tuning process, as summarized in Table 7.

**Table 7:** Hyperparameter configuration of the DNN model.

Model parameter	Selected value
1st Hidden layer neurons	384
2nd Hidden layer neurons	192
3rd Hidden layer neurons	96
Epochs	73
Batch size	32
Learning rate	4 × 10^−4^
Activation function (1st layer)	ReLU
Activation function (2nd layer)	ReLU
Activation function (3rd layer)	ReLU
Activation function (output layer)	Softmax
Optimizer	Adam
Regularization techniques	Batch normalization, dropout, ℓ_2_ regularization, and early stopping
Dropout Rates	0.20, 0.15, and 0.10
ℓ_2_ Regularization strength	1 × 10^−4^

## 5 Results

The complete dataset showed a clear class imbalance, with 1,646 Normal samples, 292 Suspect samples, and 175 Pathological samples. These correspond to 77.90%, 13.82%, and 8.28% of the dataset, respectively, indicating that the minority classes were under-represented compared with the Normal class.

Figure 11 presents the feature-level statistical relevance obtained using the Kruskal–Wallis H-test. Higher H-statistic values indicate stronger distributional differences among the Normal, Suspect, and Pathological classes. The results show that Prolonged Decelerations, Abnormal Short-Term Variability, and Percentage of Time with Abnormal Long-Term Variability had the highest H-statistic values, indicating strong discriminative ability for fetal health classification. Features such as Accelerations, Mean Value of Short-Term Variability, Histogram Mean, and Histogram Median also showed moderate relevance. In contrast, Light Decelerations, Severe Decelerations, Fetal Movement, Histogram Number of Zeroes, and Histogram Max produced lower H-statistic values, suggesting limited class-separation ability.

Table 8 presents the five most statistically significant features in the fetal health dataset, as determined by the Kruskal–Wallis H-test. This non-parametric test was used to assess whether the distribution of each numerical feature differs significantly across the three fetal health categories: Normal (1), Suspect (2), and Pathological (3). The Kruskal–Wallis test is particularly appropriate in this context because of the non-normal and skewed nature of many feature distributions, as well as the unequal sample sizes among the target classes.

**Table 8:** Top 5 most significant features based on Kruskal–Wallis test.

Feature	H-Statistic	*p*-value	Significant
Prolonged decelerations	**537.87**	1.60 × 10^−117^	True
Abnormal short term variability	525.45	7.95 × 10^−115^	True
Percentage of time with abnormal long term variability	488.67	7.69 × 10^−107^	True
Accelerations	446.13	1.33 × 10^−97^	True
Mean value of short term variability	377.10	1.30 × 10^−82^	True

Bold values indicate the features with the highest H-statistic scores, representing the strongest discriminative power across fetal health classes.

All five features shown in the table have extremely low *p*-values (≪0.05), confirming their statistical significance. Specifically:

- Prolonged Decelerations has the highest H-statistic (537.87) and an extremely low *p*-value (1.60 × 10^−117^), indicating a very strong association with fetal health outcomes.
- Abnormal Short Term Variability and Percentage of Time with Abnormal Long Term Variability also exhibit very high H-statistics and highly significant *p*-values, underscoring their clinical relevance.
- Accelerations and Mean Value of Short Term Variability are also statistically significant, supporting previous findings that variability and acceleration patterns are important markers in fetal wellbeing assessment.

Table 9 lists the five features with the lowest statistical relevance based on the Kruskal–Wallis H-test. Although these variables remained statistically significant at *p* < 0.05, their H-statistic values were comparatively lower than those of the top-ranked features reported in Table 8. This suggests that these features provide weaker separation among the fetal health classes. Therefore, during feature selection, these variables were carefully reviewed because their predictive contribution may be limited compared with features showing stronger class-discriminative ability.

**Table 9:** Least significant features based on Kruskal–Wallis test.

Feature	H-Statistic	*p*-value	Significant
Histogram width	124.08	1.14 × 10^−27^	True
Light decelerations	114.10	1.67 × 10^−25^	True
Histogram tendency	71.86	2.48 × 10^−16^	True
Histogram number of peaks	37.68	6.57 × 10^−9^	True
Histogram max	9.00	1.11 × 10^−2^	True

In this study, 11 classification models were evaluated for fetal health prediction, including nine conventional machine learning algorithms and two neural network-based models. Their performance was compared using accuracy, precision, recall, and F1-score, as summarized in Table 10. These metrics helped assess both the overall predictive performance and the ability of each model to detect clinically important minority classes, particularly the Suspect and Pathological classes.

**Table 10:** Classification report of different algorithms before cross-validation.

Sr. No	Model	Accuracy	Precision	Recall	F1-score
1	Logistic regression	87%	90%	87%	88%
2	K-Nearest Neighbors	89%	91%	89%	89%
3	Support Vector Machine	93%	93%	93%	93%
4	Decision tree	90%	90%	90%	90%
5	Random forest	95%	95%	95%	95%
6	Gradient boosting	94%	94%	94%	94%
7	XGBoost	95%	95%	95%	95%
8	LightGBM	**96%**	**96%**	**96%**	**96%**
9	AdaBoost	94%	94%	94%	94%
10	Multi-Layer Perceptron	94%	94%	94%	94%
11	DNN	92%	93%	92%	92%

Logistic Regression achieved an accuracy of 87%, with weighted-average precision, recall, and F1-score values of 90%, 87%, and 88%, respectively. This result provided a useful baseline, but it also shows that a linear model may not capture the non-linear patterns present in CTG-based fetal health data. In comparison, the SVM performed better, achieving 93% accuracy, with weighted-average precision, recall, and F1-score values of 93%.

When KNN was tested, its performance improved modestly compared with Logistic Regression. The model achieved an accuracy of 89%, with weighted-average precision and recall values of 91% and 89%, respectively, resulting in a weighted-average F1-score of 89%. This demonstrated that distance-based learning can be effective when the data are properly preprocessed and well-distributed.

The best performance was achieved by the ensemble algorithms. In particular, LightGBM obtained the highest result, with both accuracy and weighted-average F1-score reaching 96%. Random Forest and Extreme Gradient Boosting (XGBoost) also performed strongly, with both models achieving an accuracy and weighted-average F1-score of 95%. These algorithms consistently demonstrated high precision and recall, which is especially important in medical classification tasks such as fetal health prediction, where misclassifying a high-risk case can have serious consequences.

Two neural network-based algorithms were also evaluated: MLP and DNN. The MLP performed better than Logistic Regression and KNN, achieving both an accuracy and weighted-average F1-score of 94%, although it did not outperform the best ensemble method. The DNN model also showed competitive performance, achieving an accuracy of 92%, with weighted-average precision, recall, and F1-score values of 93%, 92%, and 92%, respectively. The Decision Tree model achieved an accuracy of 90%, whereas AdaBoost and Gradient Boosting achieved accuracies of 94%. Overall, the results suggest that ensemble-based algorithms, particularly LightGBM, Random Forest, and XGBoost, are highly effective for fetal health classification.

**Figure 16:**
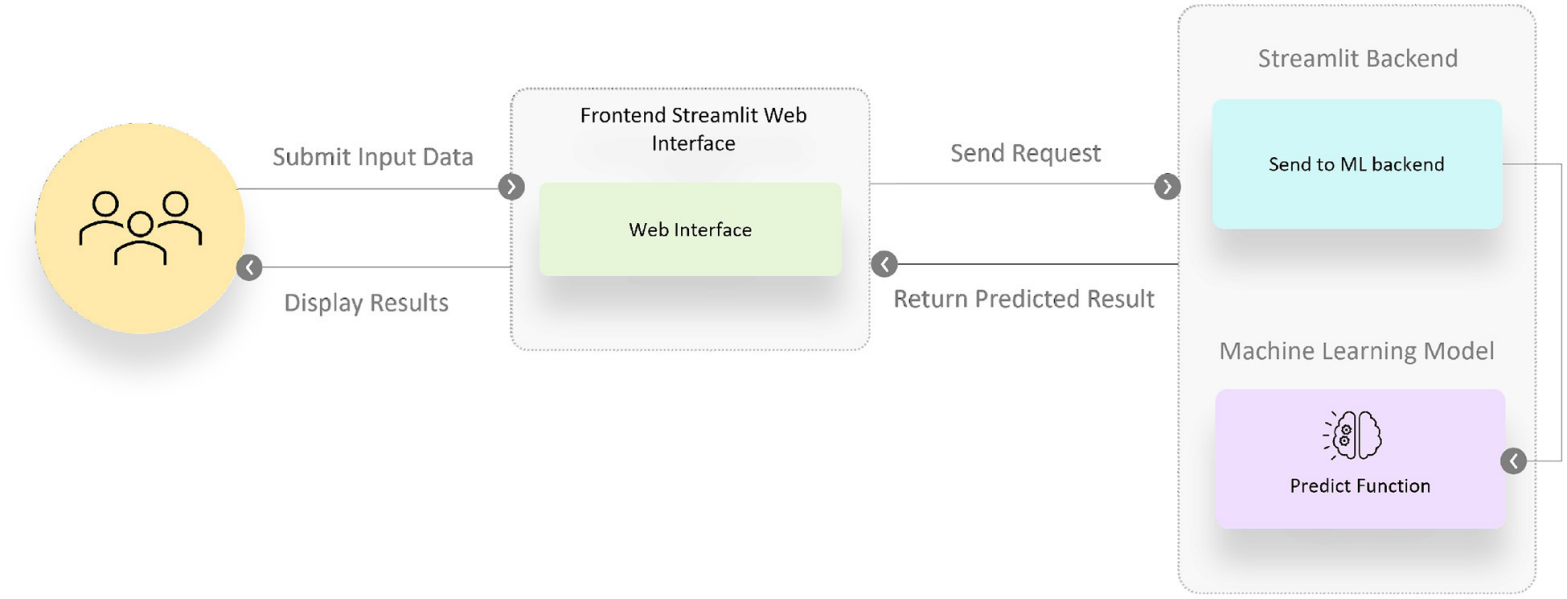
Workflow of the fetal health prediction system using Streamlit and a LightGBM classifier. The interface accepts user input, forwards it to a backend ML model, and returns the classification result to the user.

Figure 16 presents the confusion matrices of the 11 classification models evaluated for fetal health prediction: Logistic Regression, KNN, SVM, MLP, DNN, Decision Tree (DT), Random Forest (RF), AdaBoost, Gradient Boosting, LightGBM, and XGBoost. Each matrix shows the distribution of correctly and incorrectly classified samples across the three fetal health classes: Normal (Class 1), Suspect (Class 2), and Pathological (Class 3). This visualization helps identify class-wise prediction errors and provides deeper insight into how each model handles the minority classes.

**Figure 17:**
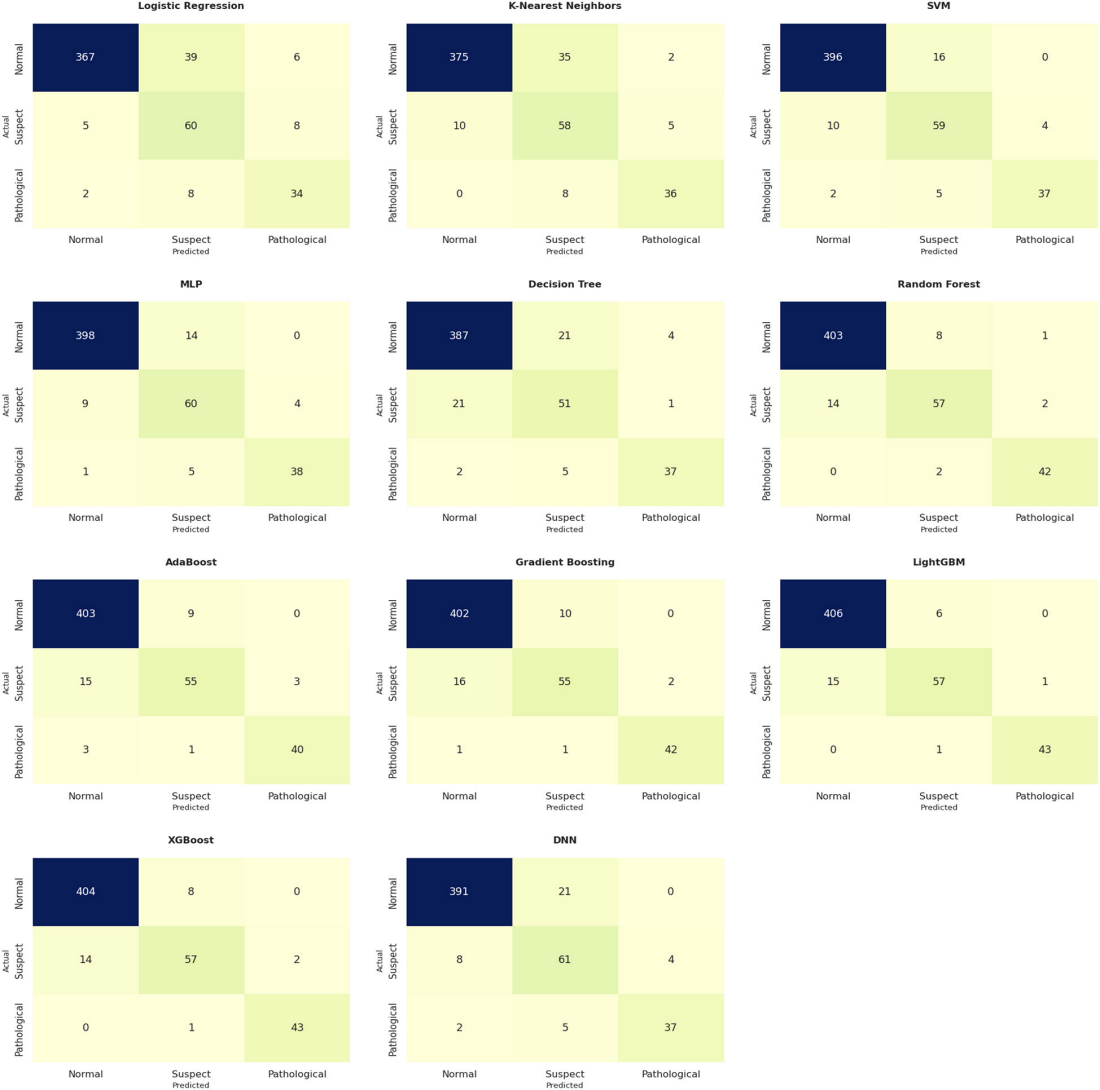
Confusion matrices of machine learning and neural network based models for fetal health classification.

Figure 17 shows the ROC curves for the three fetal health classes using a one-vs-rest evaluation approach. In this method, one class is considered positive at a time, while the remaining two classes are treated as negative. The ROC curve illustrates the trade-off between the true positive rate and the false positive rate at different threshold values. The AUC summarizes how well the model separates each class from the others, with values closer to 1 indicating stronger discriminative ability.

Table 11 presents the performance results of all 11 classifiers evaluated for fetal health classification. Logistic Regression showed the lowest performance, with an accuracy of 86.96% and a macro F1-score of 77.70%. This indicates that a linear model may not be sufficient to capture the non-linear relationships present in CTG-based fetal health features. K-Nearest Neighbors and Decision Tree performed moderately, achieving accuracies of 88.66% and 89.79%, respectively. However, the Decision Tree produced a comparatively lower AUC of 90.95%, suggesting weaker class-separation ability than the stronger models.

**Table 11:** Overall performance results of models for fetal health classification.

Model	Acc. (%)	Bal. Acc. (%)	Prec. (%)	Rec. (%)	Macro F1	AUC (%)	Kappa (%)	MCC (%)
LR	86.96%	82.39%	74.79%	82.39%	77.70%	96.20%	68.39%	69.48%
KNN	88.66%	84.10%	79.52%	84.10%	81.18%	93.95%	71.65%	72.34%
SVM	93.01%	87.01%	87.02%	87.01%	86.92%	97.29%	81.20%	81.23%
DT	89.79%	82.63%	82.91%	82.63%	82.74%	90.95%	72.39%	72.40%
RF	94.90%	90.45%	91.68%	90.45%	91.01%	98.63%	85.88%	85.92%
GB	94.33%	89.83%	91.99%	89.83%	90.87%	98.60%	84.25%	84.30%
XGB	95.27%	91.29%	92.86%	91.29%	92.00%	98.88%	86.88%	86.94%
LGBM	**96.03%**	**91.99%**	**94.36%**	**91.99%**	**93.05%**	**99.02%**	**88.91%**	**89.01%**
ADA	94.14%	88.02%	91.12%	88.02%	89.47%	96.64%	83.56%	83.66%
MLP	93.95%	88.77%	88.55%	88.77%	88.64%	98.29%	83.64%	83.65%
DNN	92.63%	87.22%	85.48%	87.22%	86.19%	97.51%	80.57%	80.70%

Bold values indicate the best performance metric achieved among all evaluated models for each column.

Among the non-ensemble models, SVM, MLP, and DNN showed better predictive performance. The DNN achieved an accuracy of 92.63% with a macro F1-score of 86.19%, while SVM obtained an accuracy of 93.01% and a macro F1-score of 86.92%. The MLP performed best within this group, reaching an accuracy of 93.95%, a macro F1-score of 88.64%, and a AUC of 98.29%. These results suggest that models capable of learning non-linear feature interactions are more effective for CTG-based fetal health classification than simpler baseline models.

The ensemble-based models showed consistently stronger performance than most standalone machine learning and neural network-based models. AdaBoost and Gradient Boosting achieved accuracies of 94.14% and 94.33%, with macro F1-scores of 89.47% and 90.87%, respectively. Random Forest further improved the results, obtaining an accuracy of 94.90% and an MCC of 85.92%. XGBoost achieved even better performance, with an accuracy of 95.27% and a macro F1-score of 92.00%, demonstrating its strong ability to model complex class boundaries.

Overall, LightGBM achieved the best performance among all evaluated models. It recorded the highest accuracy of 96.03%, balanced accuracy of 91.99%, precision of 94.36%, macro F1-score of 93.05%, AUC of 99.02%, Cohen's Kappa of 88.91%, and MCC of 89.01%. These results indicate that LightGBM provided the strongest balance between overall accuracy, minority-class recognition, and class separability, making it the most suitable model for fetal health classification in this study.

Table 12 presents the mean accuracy obtained from ten-fold cross-validation for all evaluated classifiers. LightGBM achieved the highest mean cross-validation accuracy of 97.97%, followed closely by XGBoost at 97.92% and Random Forest at 97.62%. This ranking was consistent with the independent evaluation results, where the same three ensemble models remained the top-performing algorithms.

**Table 12:** Cross-validation accuracy comparison of classification models for fetal health.

Sr. No	Model	Cross-validation accuracy
1	Logistic regression	88.33%
2	K-Nearest Neighbors	96.52%
3	SVM	97.38%
4	Decision tree	94.30%
5	Random forest	97.62%
6	Gradient boosting	97.43%
7	XGBoost	97.92%
8	LightGBM	**97.97%**
9	AdaBoost	96.43%
10	MLP	96.16%
11	DNN	92.70%

Bold values indicate the highest cross-validation accuracy achieved.

Among the leading models, LightGBM showed the smallest generalization gap of 1.94 percentage points between cross-validation and test performance, indicating stable predictive behavior across different data splits. In contrast, K-Nearest Neighbors showed the largest gap of 7.86 percentage points, reflecting its sensitivity to local data structure and class distribution. The DNN showed a very small gap of 0.07 percentage points, suggesting low performance variation; however, its overall accuracy remained lower than that of the best ensemble models. These findings indicate that cross-validation results should be interpreted together with independent test performance, as test-set evaluation provides a more conservative estimate of real-world generalization.

**Figure 18:**
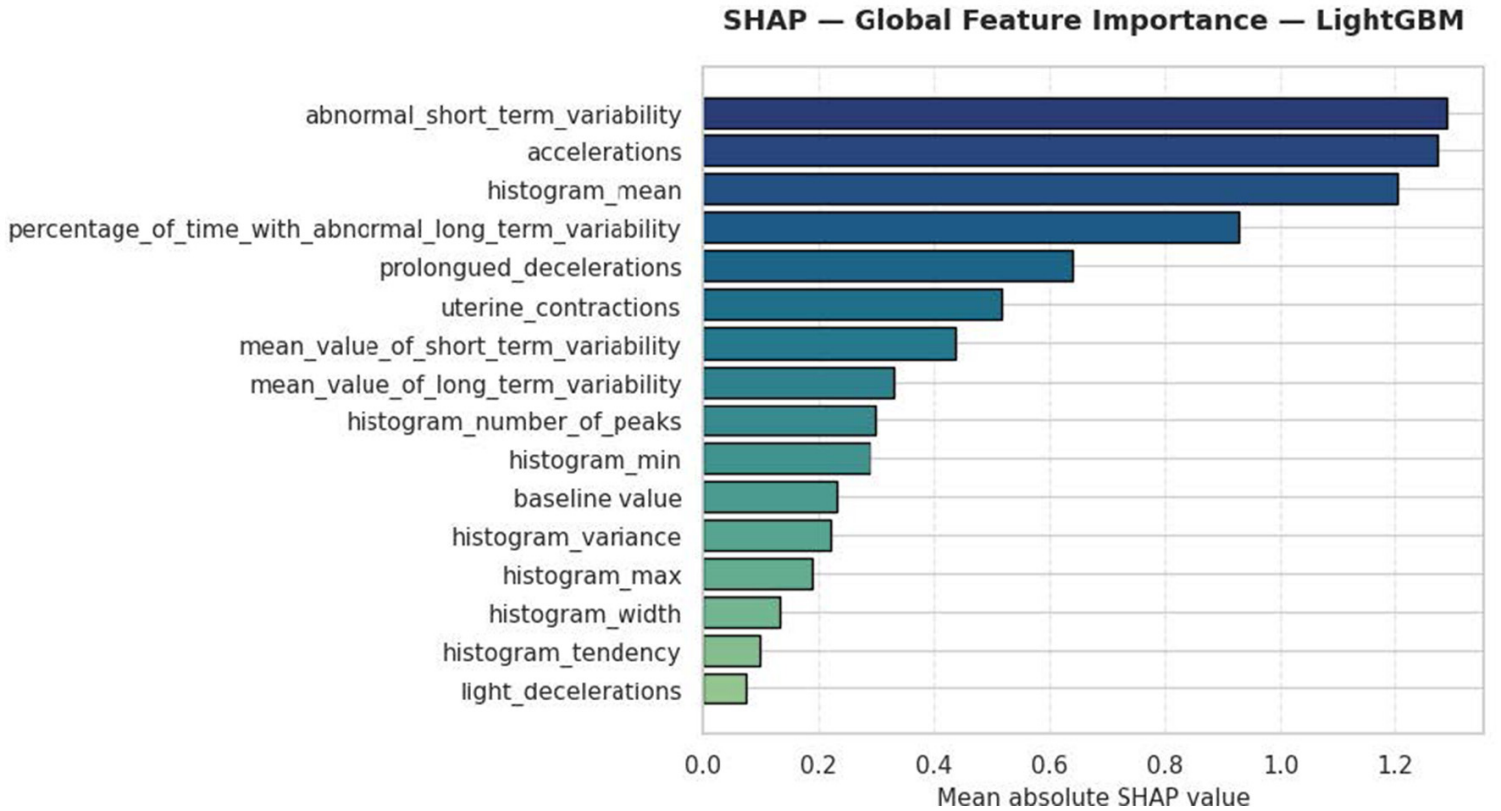
Global feature importance analysis of the LightGBM model using SHAP values.

SHAP-based global feature importance, presented in Figure 18, showed that Abnormal Short-Term Variability and Accelerations were the most influential predictors in the LightGBM model, each with a mean |SHAP| value of approximately 1.30. This indicates that abnormalities in short-term fetal heart rate variability and fetal heart rate accelerations played a major role in the model's decision-making process.

Histogram-based variables also contributed noticeably to the predictions, suggesting that the distributional characteristics of fetal heart rate signals provided useful discriminatory information. In contrast, light_decelerations showed a very small contribution, with mean |SHAP| < 0.10, indicating limited independent influence on the final classification when considered alongside other CTG-derived features.

**Figure 19:**
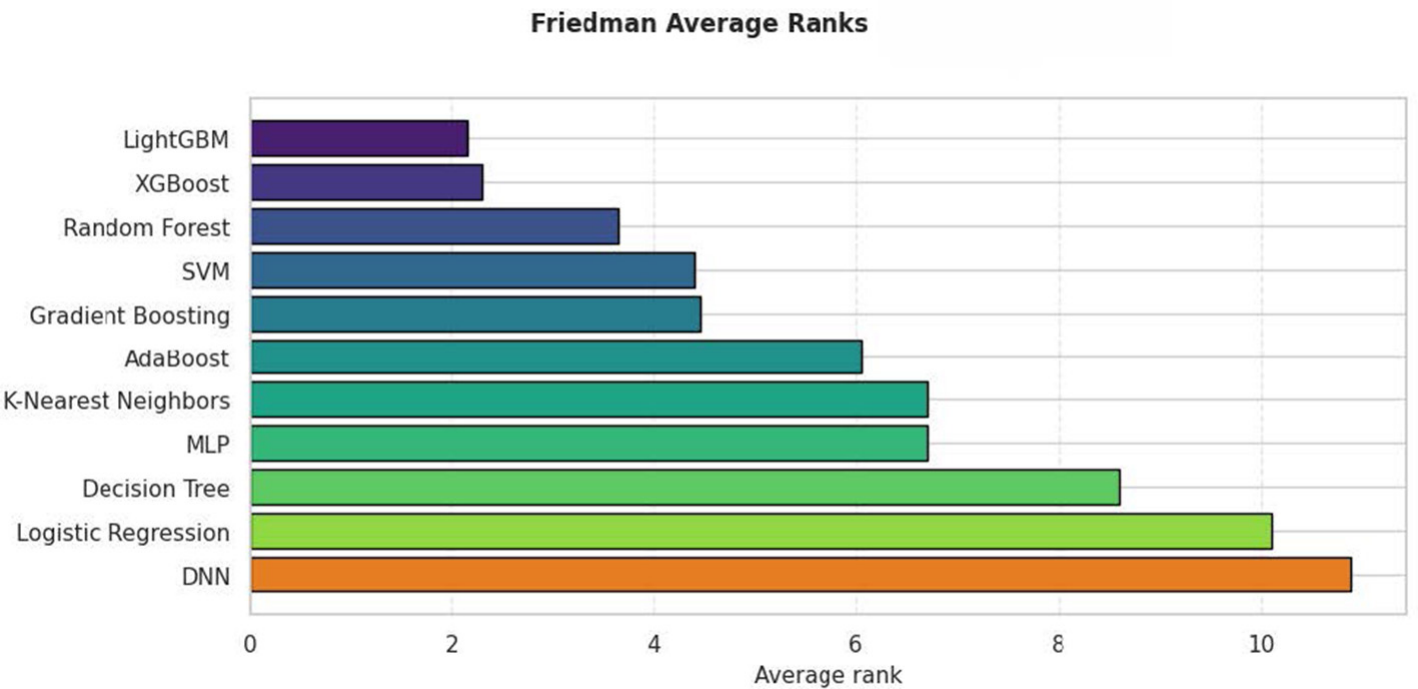
Friedman average rank comparison of the evaluated classification algorithms for fetal health prediction.

As shown in Figure 19, the Friedman test indicated a statistically significant difference in performance among the 11 evaluated classifiers (χ^2^ = 66.12, *p* < 0.001). The *post-hoc* Nemenyi test further showed that LightGBM and XGBoost achieved the best average ranks, with mean ranks of 2.2 and 2.4, respectively. Both models significantly outperformed lower-ranked classifiers such as Logistic Regression, Decision Tree, and DNN (*p* < 0.001).

No statistically significant difference was observed between LightGBM and XGBoost (*p* = 1.000). Similarly, the differences among LightGBM, XGBoost, and Random Forest were not statistically significant (*p* = 0.984 and *p* = 0.993), suggesting that these ensemble-based models formed the strongest-performing group. Although the DNN showed a very small generalization gap between cross-validation and independent evaluation accuracy, its overall performance remained lower than that of the leading ensemble models. This indicates that stable performance alone is not sufficient when the absolute predictive performance is comparatively weaker.

**Figure 20:**
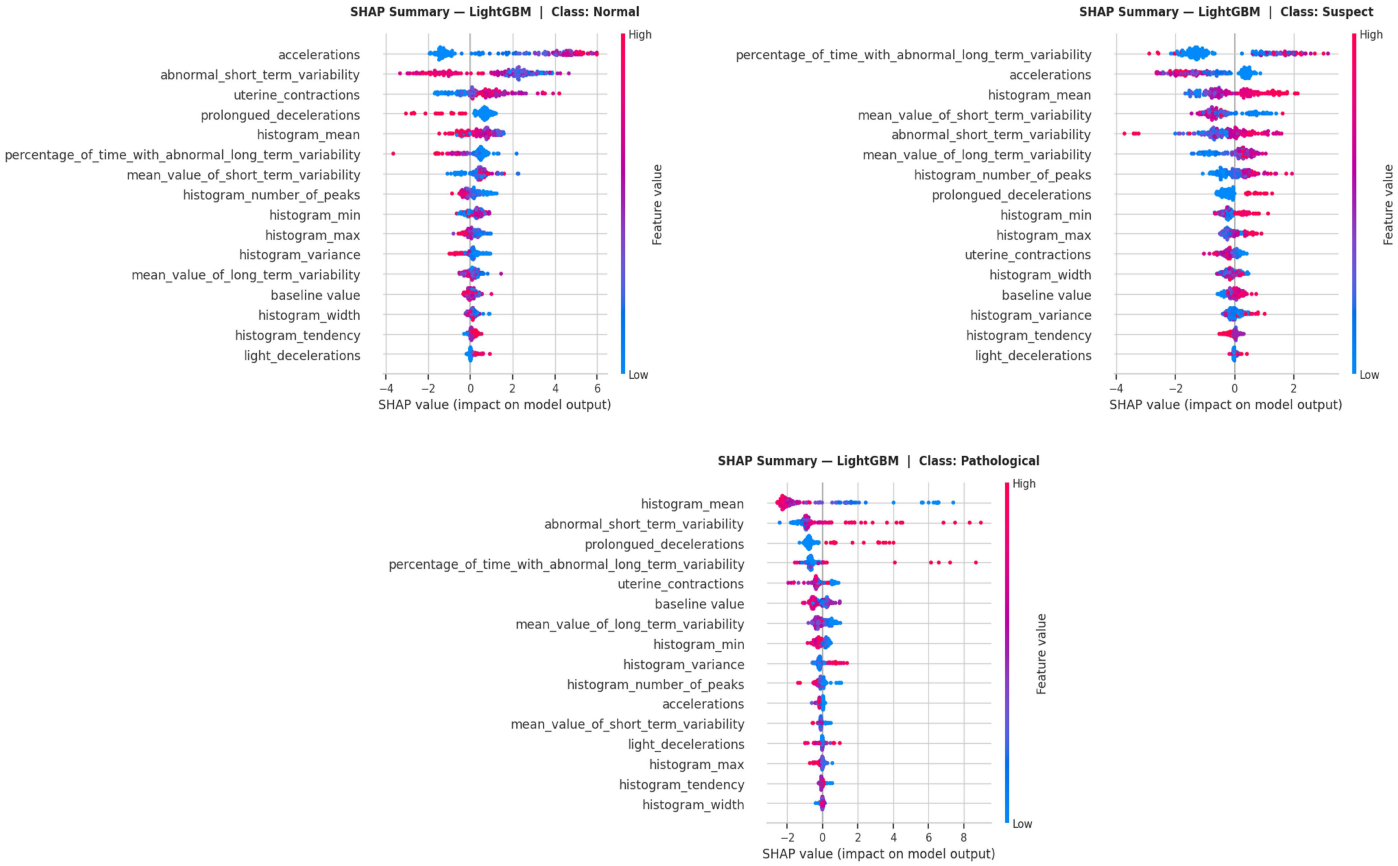
SHAP beeswarm plots of LightGBM across normal, suspect, and pathological classes.

As shown in Figure 20 the per-class SHAP beeswarm plots indicate that different feature patterns contributed to each fetal health outcome. For the Normal class, predictions were mainly influenced by higher values of Accelerations and lower influence from Prolonged Decelerations, suggesting stable fetal heart rate behavior. In the Suspect class, Percentage of Time with Abnormal Long-Term Variability showed a stronger contribution, while Accelerations had comparatively lower discriminative importance.

For the Pathological class, lower values of Histogram Mean and higher values of Abnormal Short-Term Variability were among the most influential predictors. These patterns are clinically relevant, as a reduced fetal heart rate central tendency and increased abnormalities in short-term variability may indicate fetal compromise. The SHAP values for pathological predictions reached approximately +9, indicating a stronger positive contribution toward this class than toward the Normal and Suspect classes. Interestingly, Accelerations ranked much lower for Pathological classification, suggesting that this feature contributed less to identifying severe fetal health conditions than variability- and deceleration-related features.

**Figure 21:**
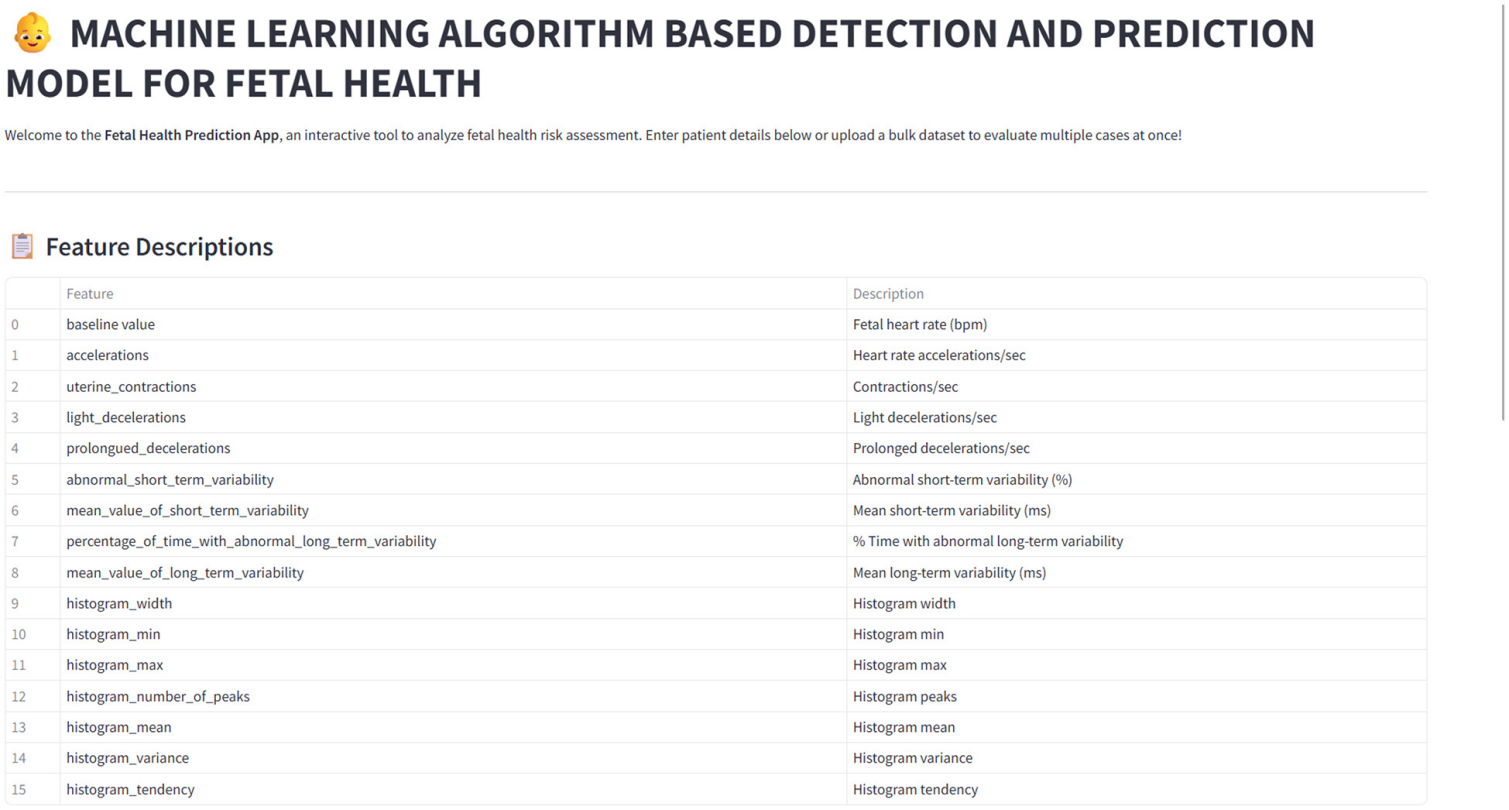
User interface of the fetal health prediction system.

Figure 21 shows the web-based prediction interface developed for fetal health classification. Through this interface, users can enter CTG-related clinical feature values and obtain the fetal health category predicted by the trained model.

**Figure 22:**
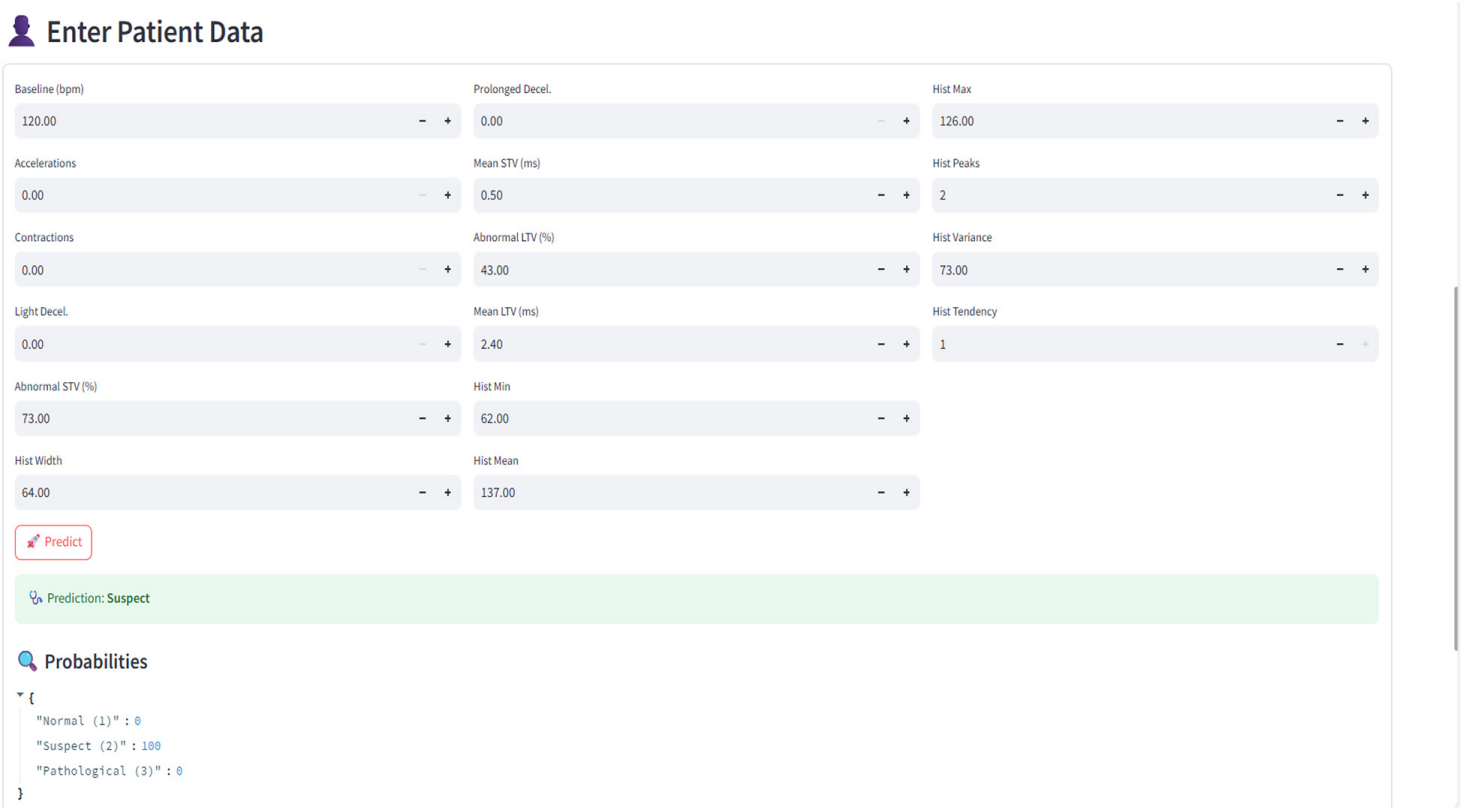
Streamlit-based web interface for real-time fetal health prediction using LightGBM model.

In this study, the final deployment model was selected based on the comparative performance analysis of all evaluated machine learning and neural network-based classifiers, as shown in Figure 22.

After evaluating the models using accuracy, balanced accuracy, precision, recall, F1-score, ROC-AUC, Cohen's kappa, MCC, and confusion matrix analysis, LightGBM was identified as the best-performing classifier. Its strong performance in distinguishing among the Normal, Suspect, and Pathological classes, along with its ability to handle non-linear feature relationships and imbalanced data effectively, made it the most appropriate model for integration into the final fetal health prediction system.

## 6 Discussion

The evaluation results reported in Table 11 show that ensemble-based and gradient-boosting models performed better than classical linear and instance-based classifiers for three-class fetal health classification using CTG features. Among all evaluated models, LightGBM achieved the strongest overall performance, with an accuracy of 96.03%, balanced accuracy of 91.99%, macro F1-score of 93.05%, ROC-AUC of 99.02%, MCC of 0.8901, and Cohen's Kappa of 0.8891. These results indicate that LightGBM was the most effective standalone classifier in this study, particularly for structured clinical tabular data where non-linear feature interactions are important.

When compared with related studies using the CTG dataset, the present results are consistent with earlier findings while also providing additional interpretability through SHAP analysis. Nazli et al. (2025) evaluated multiple classifiers, including CatBoost, ExtraTrees, LightGBM, and deep neural networks, and reported LightGBM as one of the most clinically meaningful models when balanced accuracy was considered for imbalanced CTG data. In the present study, LightGBM achieved a balanced accuracy of 91.99% under an imbalance-aware training strategy, further supporting its suitability for fetal health classification. Similarly, Innab et al. (2024) demonstrated the usefulness of LightGBM combined with SHAP-based explainability for fetal and maternal health-related prediction tasks. The present work follows a similar direction by combining strong predictive performance with per-class SHAP beeswarm analysis, allowing the model decisions to be interpreted in relation to clinically relevant CTG features.

Random Forest also showed strong performance, achieving 94.90% accuracy and 90.45% balanced accuracy, confirming the robustness of bagging-based ensemble learning. Salini et al. (2024) reported a higher Random Forest accuracy of approximately 98.45% on the same CTG dataset. The difference between that result and the present study may be attributed to variations in train–test splitting, cross-validation design, class-balancing strategy, and hyperparameter tuning. XGBoost also performed strongly, with 95.27% accuracy, 91.29% balanced accuracy, and an MCC of 0.8694. Together, LightGBM, XGBoost, and Random Forest formed the strongest group of classifiers in the present evaluation.

Previous studies on ensemble learning also support the findings of this work. Al Duhayyim et al. (2023) reported that voting-based ensemble models improved F1-score and offered better robustness than individual classifiers. Similarly, Akmal et al. (2023) and Kapila and Saleti (2023) emphasized that proper preprocessing combined with ensemble learning can improve CTG-based fetal health classification. In the present study, Gradient Boosting achieved 94.33% accuracy with a ROC-AUC of 98.60%, while AdaBoost achieved 94.14% accuracy and an MCC of 0.8366. These results follow the same trend observed in earlier studies, where ensemble methods generally performed better than single baseline models.

Among the neural network-based models, MLP achieved 93.95% accuracy and a ROC-AUC of 98.29%, making it the best non-ensemble model in this study. The DNN achieved 92.63% accuracy and a ROC-AUC of 97.51%, which was slightly lower than the MLP despite using a deeper architecture. This may be because the CTG dataset is relatively small and structured, where increasing network depth does not always improve generalization. Wong et al. (2025) applied Kolmogorov–Arnold Networks (KANs) for three-class CTG-based fetal health classification and reported 92.6% accuracy, which is close to the DNN result but lower than the LightGBM performance obtained in this study. These findings suggest that gradient-boosted tree models remain highly effective for structured CTG data while requiring lower computational complexity than deeper neural architectures. Zhao et al. (2019) also noted that deep feature extraction from raw fetal heart rate signals may require substantial computational resources, which can limit its practical use in resource-constrained clinical settings.

Among the classical machine learning models, SVM achieved 93.01% accuracy and a ROC-AUC of 97.29%, showing its ability to handle non-linear decision boundaries in high-dimensional feature spaces. This result is consistent with the work of Spilka et al. (2015), who applied SVM-based methods for intrapartum fetal heart rate analysis. In comparison, Decision Tree and K-Nearest Neighbors achieved lower accuracies of 89.79% and 88.66%, respectively. Logistic Regression recorded the lowest balanced accuracy of 82.39%, which is expected because it relies on a linear decision structure and may not fully capture the non-linear relationships present in CTG features. The Friedman test result (χ^2^ = 66.12, *p* < 0.001) further confirmed significant differences among the evaluated classifiers, while the *post-hoc* Nemenyi test ranked LightGBM and XGBoost as the top-performing models.

From a clinical evaluation viewpoint, balanced accuracy and MCC are important because the CTG dataset is dominated by the Normal class. As noted by Nazli et al. (2025), accuracy alone may hide weak detection of minority classes, especially the Pathological class, where incorrect prediction can have serious clinical consequences. In this study, LightGBM achieved an MCC of 0.8901 and Cohen's Kappa of 0.8891, indicating strong agreement between predicted and actual fetal health labels across all three classes. SHAP-based global and per-class explanations further improved the interpretability of the model, as also supported by Hamza et al. (2023) and Innab et al. (2024). Features such as abnormal short-term variability, accelerations, and histogram mean were identified as major contributors to the model's decisions, which supports the clinical relevance of the proposed framework.

## 7 Limitations and future scope

While the proposed machine learning and neural network based models have shown promising results for predicting fetal health, there are a number of limitations that could be addressed in future work.

One major limitation of this study is the usage of a single benchmark dataset namely Cardiotocography (CTG) data set obtained from UCI machine learning repository (Dua and Graff, 2019). This data set is very commonly used in research on fetal health classification, it however contains few values and has a limited structure and collected in a controlled way. CTG recordings in clinical practice might not contain complete information due to signal noise, device induced variations, missing values and population specific variations. The generalization capabilities of the model developed on this data may not be useful for clinical environment. The framework needs to be evaluated with more number of multi-center datasets to assess external validation and clinical robustness.

Another limitation is the class imbalance present in the data set, specifically, Normal and Normal classes has lesser count of occurrences. Although the SMOTEENN-based resampling has been applied to the training data set to mitigate majority class bias, the synthetic resampling technique may not be fully representative of actual complexities of clinically abnormal fetal conditions. The future work can look at cost sensitive learning, focal loss, class weighted learning, generative modeling approaches to tackle class imbalance more effectively by preserving clinically important information.

Although we tested several classical machine learning, ensemble learning, and neural network based models, the advanced neural network based models like CNNs, RNNs, LSTMs and hybrid models have not been tested extensively due to the lack of more data set in the case of structured and fewer features of the dataset. The neural network based models have the capability to capture complex spatio-temporal pattern fromraw fetal heart rate signal, which is needed to train these models from large-scale real time CTG recordings from modern fetal monitoring systems.

Temporal and longitudinal features of the fetal monitoring are not being completely used in this work. Since CTG signal are by nature time dependent and variation in the fetal heart rate over time could carry more information related to the deterioration or recovery of the fetus. Hidden Markov Models, LSTM, Gated Recurrent Units (GRUs), TCNs can be exploited to model such time dependent characteristics.

An implementation using a streamlit based web application for fetal health prediction has been developed, however this is just a prototype that is yet to be tested in a real clinical environment. Mobile based applications, integration with EHR system and involving medical experts are the key factors to be developed and analyzed on usability in real world scenarios.

Finally, issues related to AI implementation for fetal health monitoring are ethically, legally, and regulatorily complex. As stated by Leslie (2019), these can include patient data privacy, algorithmic fairness, model accountability, liability for incorrect prediction etc. Thus, this future work should focus on privacy preserving AI through Federated learning, model updates, bias aware AI and explainable AI.

## 8 Conclusion

This research paper proposes a structured machine learning and neural network pipeline for the classification of the health status of the fetus according to the derived attributes from CTG records. The entire process includes data cleansing, normalization of features, performing statistical tests, class rebalancing by means of SMOTEENN technique, estimation of multicollinearity in terms of Variance Inflation Factor (VIF), feature selection based on Kruskal-Wallis hypothesis test, and evaluation of models based on various performance measures. In the scope of classifiers, the LightGBM algorithm achieved the best results, providing an accuracy score of 96.03%, balanced accuracy score of 91.99%, macro F1-score of 93.05%, ROC-AUC score of 99.02%, Cohen's Kappa coefficient of 0.8891, and MCC score of 0.8901. Other ensemble learning techniques such as XGBoost, Random Forest, and Gradient Boosting have also yielded reliable outcomes, thus confirming that ensemble models are effective when dealing with structured clinical classification tasks. Moreover, SHAP analysis showed that the predictors with the largest impact were abnormal short-term variability and accelerations, reflecting the importance of these variables from a clinical standpoint.

Furthermore, the developed machine learning model was used to build a web-based predictor. The developed application allows users to enter attribute values related to CTG and get the predicted status of fetal health. While currently presented only as a prototype, this technology can potentially be used in the creation of tools for assisting clinicians in the assessment of fetal wellbeing through machine learning models.

To extend the study, further work should focus on verifying the suggested framework on a greater number of clinical datasets gathered from multiple centers. Also, temporal neural network based models, for instance, LSTM and GRU, can be explored in order to develop hybrid models for analyzing raw or sequential CTG signals. Other directions of research might include feature selection techniques, Explainable AI (XAI) methods, developing mobile applications, and integrating the system with the EHR. From an ethical perspective, issues of consent, privacy, and other regulations need to be taken into account prior to implementation in clinical settings.

## Data Availability

The dataset used in this study is the publicly available CTG dataset from the UCI Machine Learning Repository (https://archive.ics.uci.edu/dataset/193/cardiotocography), which is freely accessible.
